# The polyamino‐isoprenyl enhancer NV716 enables the antibacterial activity of two families of multi‐target inhibitors against the ESKAPEE bacterium *Enterobacter cloacae*

**DOI:** 10.1002/mlf2.70014

**Published:** 2025-06-25

**Authors:** Emma Forest, Jordan Lehoux, Alexandre Guy, Thierry Durand, Stéphane Audebert, Luc Camoin, Christopher D. Spilling, Céline Crauste, Stéphane Canaan, Jean Michel Brunel, Jean‐Michel Bolla, Jean‐François Cavalier

**Affiliations:** ^1^ Aix Marseille Univ., CNRS, LISM UMR7255, IMM FR3479 Marseille France; ^2^ Aix Marseille Univ, INSERM, SSA, MCT Marseille France; ^3^ IBMM, Univ Montpellier, CNRS, ENSCM Montpellier France; ^4^ Aix Marseille Univ, CNRS, INSERM, Institut Paoli‐Calmettes, CRCM, Marseille Protéomique Marseille France; ^5^ Department of Chemistry and Biochemistry University of Missouri‐St. Louis St. Louis Missouri USA

**Keywords:** activity based‐protein profiling, antibiotic resistance, Cyclipostins and Cyclophostin analogs, drug enhancers, oxadiazolone derivatives

## Abstract

Gram‐negative bacteria are particularly prone to developing antimicrobial resistance (AMR), as evidenced by the WHO's ESKAPEE list of high‐priority pathogens. One strategy that has increased is the use of antibiotic enhancers, which can re‐empower abandoned or poorly active antibiotics against the resistant strain of interest. In this study, the polyamino‐isoprenyl antibiotic enhancer, NV716, was tested in combination with two families of multi‐target Ser/Cys‐based enzyme inhibitors, the oxadiazolone derivatives (OX) and the Cyclipostins and Cyclophostin analogs (CyC), which are inactive against Gram‐negative ESKAPEE bacteria, to potentiate their antibacterial activity and thus make them active against these bacteria. We demonstrated that NV716 potentiates some OX and CyC compounds by permeabilizing the outer membrane and thus by increasing the inhibitor accumulation, as shown by fluorescence microscopy. By using the click‐chemistry activity‐based protein profiling (ABPP) approach coupled with proteomic analysis, we also confirmed the multi‐target nature of the best OX and CyC inhibitors by identifying their target proteins on a bacterial culture of *Enterobacter cloacae*. Remarkably, a large set of these identified proteins had already been captured in previous ABPP experiments conducted on *Mycobacterium tuberculosis* and/or *Mycobacterium abscessus* culture. Furthermore, we showed that five of the identified target proteins were present in a total lysate of *Pseudomonas aeruginosa*. Importantly, these latter enzymes are highly conserved among Gram‐negative bacteria, with two of them annotated as essential for bacterial survival. These results provide proof of concept that both OX and CyC, if successfully potentiated, could be used against ESKAPEE Gram‐negative bacteria.

## Impact statement

The increasing incidence of resistant Gram‐negative bacteria and the lack of new drugs underscore the urgent need for new antimicrobial agents. A promising strategy to overcome this problem is the use of antibiotic adjuvants, which can revive poorly active antibiotics against resistant strains of interest. Here, we report the potentiating effect of the polyamino‐isoprenyl adjuvant NV716 in activating the antimicrobial activities of two families of multi‐target inhibitors, initially ineffective on Gram‐negative bacteria, against the ESKAPEE bacterium *Enterobacter cloacae*. The mechanism of action of NV716 and the potential target enzymes of our inhibitors in *En. cloacae* were also elucidated, highlighting the antibacterial potential of our inhibitors against bacterial pathogens.

## INTRODUCTION

The discovery and development of antimicrobials, such as penicillin, during the 20th century revolutionized chemotherapy to a point where the healthcare community believed that the war against microbes had been won^1^, ^2^. In fact, between the 1940s and the 1960s, the scientific community experienced what is now known as the “Golden Era” of antibiotics, during which a large number of new drugs were developed, but since then, very few new classes have been discovered. However, while antibiotics have strongly transformed our relationship with disease‐causing bacteria, empirical prescription in the case of infections contributed greatly to the emergence of antibiotic resistance among bacterial populations. The spread of antimicrobial resistance (AMR), coupled with the decline in antibiotic development, has become a major public health concern. Recent studies have estimated that infections caused by multidrug‐resistant (MDR) bacteria are responsible for 700,000 deaths each year^3^. Another statistical model estimated that nearly 5 million deaths were associated with AMR in 2019^4^. To refocus the scientific community's efforts on this issue, in 2017, the WHO regrouped the highest‐priority pathogens with respect to AMR into the ESKAPEE list^5^, ^6^, which includes two Gram‐positive bacteria: *Enterococcus faecium* and *Staphylococcus aureus*, and five Gram‐negative bacteria, *Klebsiella pneumoniae*, *Acinetobacter baumannii*, *Pseudomonas aeruginosa*, *Enterobacter* spp., and *Escherichia coli*. Among these, Gram‐negative bacteria are particularly overrepresented. This is due to their high propensity to develop many resistance mechanisms^7^, ^8^, in addition to their intrinsic resistance to many antimicrobials resulting from their membrane composition^9^, ^10^, and the expression of broad‐spectrum efflux pumps^11^. In fact, the efflux pumps may act synergistically with the impermeability of the outer membrane to increase antibiotic resistance. As a direct consequence, some Gram‐negative antibiotics may become clinically obsolete.

**Figure 1:**
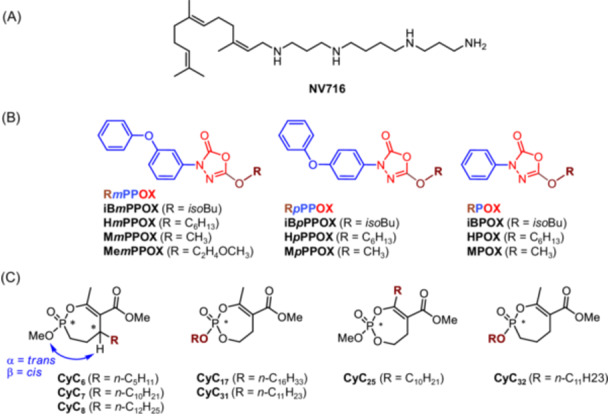
Chemical structures of compounds. (A) Polyamino‐isoprenyl enhancer NV716^17^, ^18^. (B) Oxadiazolone‐core derivatives^19^, ^20^. (C) The Cyclipostins and Cyclophostin analogs^21^, ^22^, ^23^.

With a significant slowdown in the development of new antimicrobials, largely due to a poor return on investment^1^, new strategies to overcome MDR need to be explored. The concept of drug adjuvants, also called “adjuvants,” “chemosensitizers,” or “antibiotics enhancers,” able to (re)activate poorly active antibiotics against resistant strains, constitutes a real valuable strategy^12^, ^13^. These adjuvants are chemical entities that have no intrinsic antimicrobial activity, but can potentiate/activate inefficient antibiotics, either by protecting the drug from enzymatic degradation (i.e., β‐lactamase inhibitors^14^) or by enhancing its efficacy through membrane permeabilization or efflux pump blockade^13^, ^15^, ^16^. In this context, we have demonstrated that the polyamino‐isoprenyl derivative NV716 (Figure 1A) was able to restore the activity of disused antibiotics, such as doxycycline, chloramphenicol, and rifampicin, against *P. aeruginosa* MDR strains^24^, including clinical isolates from cystic fibrosis (CF) patients^13^, ^25^. Its enhancing properties resulted from an increase in the outer‐membrane permeabilization as well as the inhibition of efflux mechanisms^24^, ^26^.

Some antibiotics, such as polymyxin B, which permeabilizes both the outer and the inner membrane of Gram‐negative bacteria, or ciprofloxacin, which inhibits topoisomerases II and IV, are known to exert multiple mechanisms of action^27^, and their use in combination therapy is frequent when dealing with MDR bacteria^28^, ^29^. Accordingly, another valuable strategy is the development of multi‐target compounds to prevent the emergence of target‐specific resistant strains^27^, thus simplifying polytherapy treatment for the patient. In this challenging context, a few years ago, we have developed two families of multi‐target inhibitors: the oxadiazolone‐core derivatives (OX) and the Cyclipostins and Cyclophostin analogs (CyC) (Figure 1B,C)^30^. These compounds bind covalently to catalytic serine or cysteine residues in the active site of target proteins, thereby inhibiting the enzymatic activity of corresponding (Ser/Cys)‐based enzymes^19^, ^21^. Nontoxic to mammalian host cells, both OX and CyC specifically impair the growth of all mycobacteria, including *Mycobacterium abscessus* ^22^, ^31^ and *Mycobacterium tuberculosis* ^20^, ^32^, in broth medium and/or within infected macrophages. Using activity‐based protein profiling (ABPP) approaches on *M. abscessus* ^22^, ^31^ and *M. tuberculosis* ^20^, ^32^, ^33^ cultures, we clearly demonstrated the multi‐target inhibitory activity of these compounds by identifying all their target mycobacterial enzymes. Despite the huge number of (Ser/Cys)‐based enzymes in all prokaryotic cells, the OX and CyC are, however, not active against Gram‐negative bacteria^34^. Using fluorescent CyC analogs, we unambiguously confirmed that this lack of antibacterial activity was not due to the absence of targets, but rather due to the inability of these molecules to cross the outer membrane of such bacteria^35^. Indeed, when treating a total lysate of *P. aeruginosa* with the same fluorescent CyC inhibitors, distinct fluorescent bands were detected on SDS‐PAGE gel corresponding to bacterial (Ser/Cys)‐based enzymes that may have reacted with our inhibitors^35^.

From these findings, it is thus tempting to assume that if OX and CyC could penetrate the outer membrane of Gram‐negative bacteria, they would be able to block various metabolic pathways impacting their growth, as in the case of *M. abscessus* or *M. tuberculosis*. Therefore, in this study, we evaluated the efficiency of the OX and CyC in association with the polyamino‐isoprenyl enhancer NV716, against a panel of four Gram‐negative ESKAPEE bacteria: *P. aeruginosa*, *K. pneumoniae, E. coli,* and *Enterobacter cloacae*. Of the four bacteria tested, only *En. cloacae* was sensitive to OX/CyC–NV716 combinations, with significant MIC values obtained. A series of real‐time assays (permeabilization and/or disruption of the outer membrane, depolarization of the inner membrane, inhibition of efflux pumps) combined with quantitative fluorescence microscopy experiments helped to elucidate the mechanism of action by which NV716 potentiates the activity of OX and CyC in *En. cloacae*. The potential targets of the two most potent OX and CyC inhibitors were further identified by click‐chemistry ABPP (CC‐ABPP) using their corresponding click‐ready affinity‐based probes on a bacterial culture of *En. cloacae* in the presence of NV716. The same approach conducted on a *P. aeruginosa* lysate revealed similar target enzymes, suggesting that, provided they can cross the outer membrane, the OX and CyC inhibitors could represent an interesting therapeutic alternative against resistant Gram‐negative bacteria.

## RESULTS

### The polyamino‐isoprenyl derivative NV716 enables the antibacterial activity of the OX and CyC compounds against *En. cloacae* ATCC 23355

The antibacterial properties of selected OX and CyC active on *M. abscessus* and *M. tuberculosis* growth (i.e., 7 OXs and 9 CyCs; Figure 1B,C and Table 1)^30^ have been tested in the presence or absence of NV716 against four Gram‐negative bacteria belonging to the ESKAPEE group: *P. aeruginosa* PAO1, *K. pneumonia* ATCC 13883, *E. coli* ATCC 25922, and *En. cloacae* ATCC 23355. Their corresponding minimal inhibitory concentrations leading to 90% of growth inhibition, hereinafter referred to as MIC_90_, determined by the rapid p‐iodonitrophenyltetrazolium violet (INT) colorimetric assay following absorbance reading at 470 nm^36^, ^37^, are shown in Table 1. As expected, when tested alone, OX and CyC were fully inactive against the four Gram‐negative bacteria (MIC_90_ > 100 µg/ml). To exclude any indirect growth defect caused by the polyamino‐isoprenyl derivative, NV716 was used at a fixed sub‐MIC concentration of 4.1 µg/ml (i.e., 0.2× MIC_90_ – MIC__NV716_ = 20.3 µg/ml) with *P. aeruginosa* PAO1 and *K. pneumonia* ATCC 13883^24^, and at 1.2 µg/ml (i.e., 0.24× MIC_90_ – MIC_90_NV716_ = 5.0 µg/ml) with *E. coli* ATCC 25922^24^ and *En. cloacae* ATCC 23355 for the combination assays with the OX and CyC compounds (Figure S1). Such sub‐MIC concentrations have previously been validated through synergistic assays and subsequent determination of the Fractional Inhibitory Concentration Index (FICI) of various antibiotics in combination with NV716 against these Gram‐negative bacteria^38^, ^39^, ^40^.

**Table 1:** Antibacterial activities of selected OX and CyC in the presence or absence of NV716 against four Gram‐negative bacteria belonging to the ESKAPEE group^a^.

	MIC_90_ (µg/ml)									
			*En. cloacae* ATCC 23355		*E. coli* ATCC 25922		*P. aeruginosa* PAO1		*K. pneumonia* ATCC 13883	
Cpds	*M. tuberculosis* mc²6230^b^	*M. abscessus* CIP 104536^T^ S^c^	− NV716	+ NV716 (1.2 µg/ml)^d^	−NV716	+NV716 (1.2 µg/ml)^d^	− NV716	+ NV716 (4.1 µg/ml)^d^	− NV716	+ NV716 (4.1 µg/ml)^d^
**iBPOX**	>100	16 ± 0.56	>100	80 ± 14.2	>100	>100	>100	>100	>100	>100
**iB** ***p*** **PPOX**	22 ± 0.70	28 ± 1.8	>100	>100	>100	>100	>100	>100	>100	>100
**HPOX**	43 ± 0.80	26 ± 1.4	>100	80 ± 16.3	>100	>100	>100	>100	>100	>100
**H** ***p*** **PPOX**	20 ± 0.8	28 ± 1.2	>100	>100	>100	>100	>100	>100	>100	>100
**M** ***m*** **PPOX**	68 ± 2.9	34 ± 1.2	>100	>100	>100	>100	>100	>100	>100	>100
**M** ***p*** **PPOX**	>100	45 ± 1.8	>100	>100	>100	>100	>100	>100	>100	>100
**Me** ***m*** **PPOX**	39 ± 1.1	15 ± 0.65	>100	75 ± 20.4	>100	>100	>100	>100	>100	>100
**CyC**_**6α**_	53 ± 2.3	>100	>100	>100	>100	>100	>100	>100	>100	>100
**CyC**_**6β**_	>100	65 ± 1.7	>100	>100	>100	>100	>100	>100	>100	>100
**CyC**_**7α**_	>100	79 ± 1.8	>100	19 ± 6.2	>100	>100	>100	>100	>100	>100
**CyC**_**7β**_	14 ± 1.5	73 ± 1.8	>100	19 ± 6.2	>100	>100	>100	>100	>100	>100
**CyC**_**8β**_	>100	74 ± 1.7	>100	>100	>100	>100	>100	>100	>100	>100
**CyC**_**17**_	1.2 ± 0.03	6.5 ± 0.12	>100	>100	>100	>100	>100	>100	>100	>100
**CyC**_**25**_	42 ± 1.5	34 ± 0.31	>100	19 ± 5.1	>100	>100	>100	>100	>100	>100
**CyC**_**31**_	2.0 ± 0.06	0.44 ± 0.11	>100	>100	>100	>100	>100	>100	>100	>100
**CyC**_**32**_	2.0 ± 0.26	63 ± 0.74	>100	6.5 ± 2.0	>100	>100	>100	>100	>100	>100
**DOX** ^e^	‐	‐	7.5 ± 0.3	0.16 ± 0.01	2	0.031	16	1	1.1 ± 0.9	1.2 ± 0.7
**CHL** ^e^	‐	‐	11 ± 0.6	1.7 ± 0.6	8	0.5	32	0.125	2.5 ± 1.5	2.0

^a^Experiments were performed as described in the Materials and Methods section. MIC_90_, compound minimal concentration leading to 90% of growth inhibition, as determined by the p‐iodonitrophenyltetrazolium violet (INT) assay following absorbance reading at 470 nm. Values are mean of at least three independent assays performed in triplicate. ^b^Data from Refs.^20^, ^32^, ^33^, ^34^. ^c^Data from Refs.^22^, ^31^, ^34^, ^35^. ^d^The final concentration of the enhancer is indicated under parenthesis. ^e^Data on *E. coli* ATCC 25922, *P. aeruginosa* PAO1, and *K. pneumoniae* ATCC 13883 are from Refs.^24^, ^26^. CHL, chloramphenicol; DOX, doxycycline; INT, p‐iodonitrophenyltetrazolium violet.

In these conditions, and among the four bacteria tested, NV716 was able to potentiate the activity of 3 OXs and 4 CyCs against *En. cloacae* ATCC 23355 only. If the OX–NV716 association resulted in weak antimicrobial activity (MIC_90_ = 75–100 µg/ml), significant and promising MIC_90_ values were obtained with the four CyC–NV716 associations (Table 1). Interestingly, CyC_**17**_ and CyC_**31**_, which showed the best antibacterial activity against mycobacterial growth, did not affect *En. cloacae* ATCC 23355 even in the presence of the drug enhancer NV716. Conversely, NV716‐potentiated CyC_**7α,β/25/32**_ showed MIC_90_ values in the same range as the reference drugs doxycycline (7.5 ± 0.3 µg/ml) and chloramphenicol (11 ± 0.6 µg/ml) when tested alone.

We further evaluated the efficiency of the OX and CyC combination with NV716 in the context of antimicrobial resistance by testing the susceptibility of five clinical *En. cloacae* strains collected in three hospital centers located in the south of France between October 2017 and May 2020 and characterized in a previous study (Table S1)^41^. First, all five clinical *En. cloacae* strains were almost 16 times less susceptible to the effect of NV716 (MIC_90_*Ecl*‐isolate_ = 80 µg/ml) than the reference strain ATCC 23355 (MIC_90_ = 5 µg/ml). Remarkably, the presence of only 1.2 µg/ml (= 0.015× MIC_90_*Ecl*‐isolate_) NV716 was sufficient to potentiate the antibacterial activity of doxycycline and chloramphenicol by at least 2.4‐fold and up to 18‐fold in the case of *Ecl71* (Table S2). Interestingly, *Ecl136* remained insensitive to chloramphenicol (MIC_90_ > 64 µg/ml) even in the presence of NV716.

Drug susceptibility testing of the most active compounds, iBPOX and CyC_**32**_, against the five *En. cloacae* clinical isolates in the presence or absence of NV716 was further investigated (Table 2). With iBPOX, although no MIC_90_ was achieved for any clinical isolate despite the addition of NV716 up to 20 µg/ml (= 0.25 × MIC_90_*Ecl*‐isolate_), the same iBPOX–NV716 association was able to inhibit 50% of the growth of most isolates at concentrations (MIC_50_ = 37.8–92.8 µg/ml), similar to the one obtained for the reference strain ATCC 23355 (MIC_50_ = 44.9 µg/ml) (Table 2). On the other hand, the CyC_32_–NV716 association displayed good MIC_50_ and MIC_90_ (Table 2) values against *Ecl71* (MIC_50/90_ = 10.0 ± 1.9/39.1 ± 14.5 µg/ml) and *Ecl93* (MIC_50/90_ = 5.3 ± 1.6/9.7 ± 4.5 µg/ml), similar to those obtained with reference drugs (Table S2).

**Table 2:** Antibacterial activities of selected OX and CyC in the presence or absence of NV716 against *En. cloacae* clinical isolates^a^.

	MIC_50_/MIC_90_ (µg/ml)			
	iBPOX		CyC_32_	
*En. cloacae* strains	−NV716	+NV716 (20 µg/ml)	−NV716	+NV716 (20 µg/ml)
**ATCC 23355**	>100	44.9 ± 4.3/80.0 ± 14.2	>100	3.7 ± 0.26/6.5 ± 2.0
***Ecl53***	>100	92.8 ± 1.4/>100	>100	>100
***Ecl71***	>100	70.1 ± 14.3/>100	>100	10.0 ± 1.9/39.1 ± 14.5
***Ecl80***	>100	>100	>100	>100
***Ecl93***	>100	>100	>100	5.3 ± 1.6/9.7 ± 4.5
***Ecl136***	>100	37.8 ± 17.9/>100	>100	>100

^a^ Experiments were performed as described in the Materials and Methods section. MIC_50_/MIC_90_, compound minimal concentration leading to 50% and 90% of growth inhibition, respectively, as determined by the INT assay following absorbance reading at 470 nm. Values are means of at least three independent assays.

Taken together, these results underscore the potentiating effect of NV716 in activating the OX and CyC antibacterial activity against the *En. cloacae* ATCC 23355 reference strain as well as clinical isolates.

### The CyC_32‐Dansyl_ is able to label *En. cloacae* ATCC 23355 only in the presence of NV716

**Figure 2:**
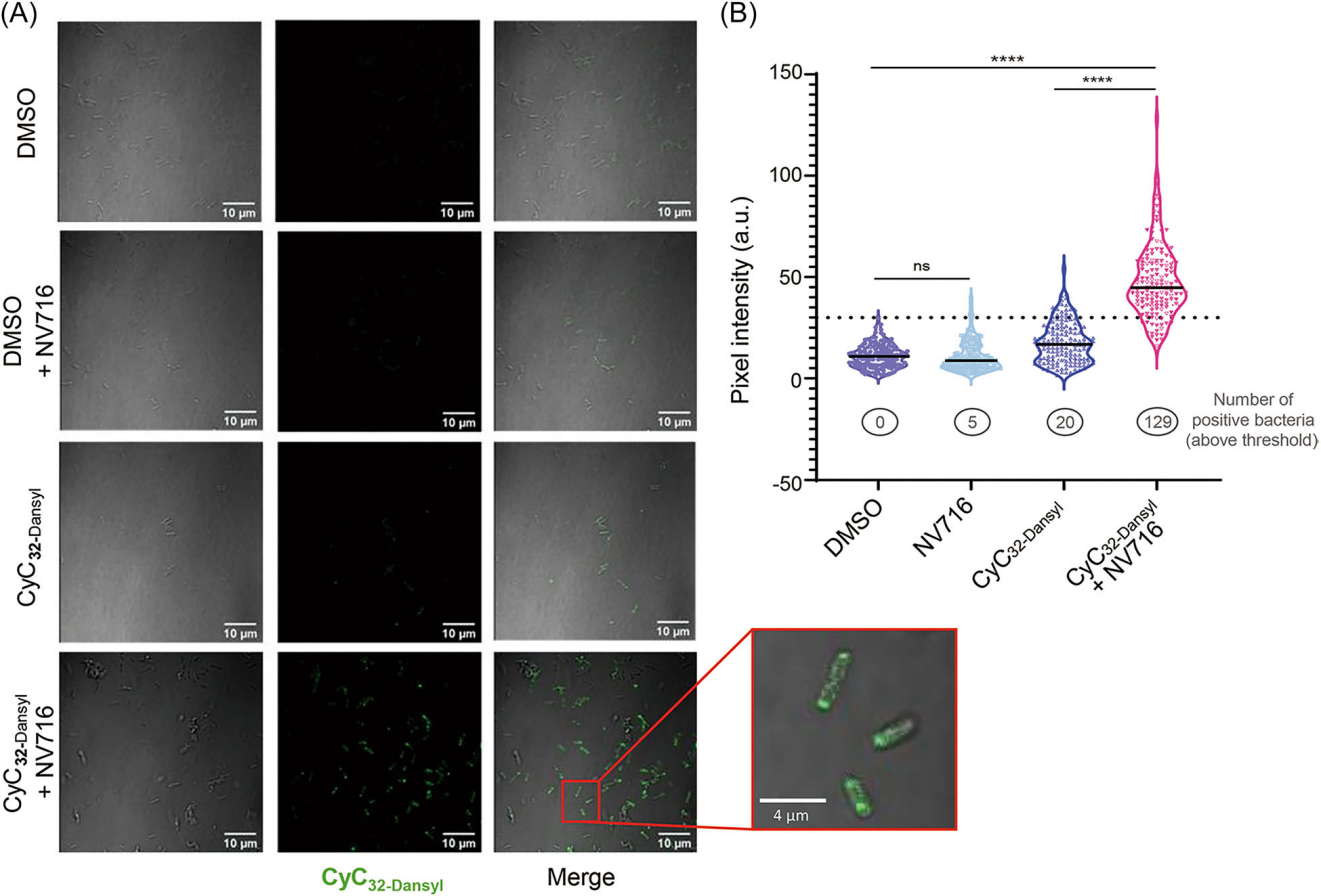
NV716 allows fluorescent compound CyC_32‐Dansyl_ to accumulate in *Enterobacter cloacae* ATCC 23355. (A) Representative fluorescence images of the *En. cloacae* ATCC 23355 strain in the presence of fluorescent CyC_32‐Dansyl_. The bacteria were exposed to 124 μg/ml CyC_32‑Dansyl_ or DMSO (vehicle, negative control) in the presence or absence of 40.6 µg/ml NV716, washed, and fixed with 4% paraformaldehyde. Fixed bacteria were imaged using an Olympus IX81 confocal microscope^35^. Scale bars: 10 μm. (B) Quantitative analysis of the Dansyl fluorescence signal per bacterium shown as a violin plot and expressed as pixel intensity arbitrary units (a.u.). To eliminate any false‐positive hits due to bacterial autofluorescence, the maximum fluorescence intensity recovered with the vehicle (DMSO only) was used as the threshold. All CyC_32‐Dansyl_‐treated bacteria with a fluorescence signal above this value (i.e., values above the dotted line) were therefore considered probe‐positive. The number under each violin represents the number of “positive” bacteria counted above this threshold in each condition. Results are from three biologically independent experiments, *n *= 150, for each condition. The *p*‐values were calculated using a two‐tailed *t*‐statistic test with Prism 8.0 (Graphpad, Inc). ****p‐value < 0.0001; ns, not significant (*p*‐value > 0.05).

The ability of NV716 to potentiate the iBPOX and CyC_32_ compounds may result from better penetration of these two inhibitors into the bacteria following disruption/permeabilization of the *En. cloacae* outer membrane by the adjuvant. To confirm this hypothesis, *En. cloacae* ATCC 23355 was preincubated with the CyC_32‑Dansyl_ fluorescent probe in the presence or absence of NV716 and the obtained CyC_32‑Dansyl_‐treated bacteria were further processed for fluorescent microscopy, as previously reported^35^. In the absence of NV716, only a slight and diffuse fluorescence was detected, suggesting that the CyC_32‐Dansyl_ alone was not able to cross the *En. cloacae* membrane. On the contrary, when incubated in the presence of NV716, this fluorescent CyC probe was found to accumulate inside the bacteria. Interestingly, this compound seems to localize as foci closed to the bacterial pole (Figure 2A—inserted zoom). Quantitative analysis of CyC_32‑Dansyl_ accumulation in *En. cloacae* ATCC 23355 was next performed (Figure 2B). In the presence of NV716, a significantly higher level of CyC_32‐Dansyl_ associated with *En. cloacae* was reached rather than in the absence of the polyamino‐isoprenyl adjuvant (average mean pixel intensity [a.u.] of 47.9 ± 1.5 vs. 17.6 ± 0.8, for CyC_32‐Dansyl_ + NV716 positive bacteria vs. CyC_32‐Dansyl_ positive bacteria, respectively, *p*‐value < 0.0001) (Figure 2B). Conversely, incubation of *P. aeruginosa* PAO1 with CyC_32‐Dansyl_ in the presence of NV716 did not result in any fluorescence uptake (Figure S2A). Indeed, no significant differences in the quantitative fluorescence signal were observed (average mean pixel intensity [a.u.] of 26.3 ± 1.4 vs. 24.3 ± 2.4, for CyC_32‐Dansyl_ + NV716 vs. DMSO, respectively) (Figure S2B), indicating that NV716 is not able to potentiate CyC_32‐Dansyl_ penetration inside *P. aeruginosa*.

### NV716 acts through permeabilization of the outer membrane and depolarization of the inner membrane of *En. cloacae* ATCC 23355

A series of real‐time assays have been previously reported^13^, ^17^, ^24^, ^40^ and used to determine the capacity of NV716, tested at two working concentrations of 6 and 50 µg/ml (i.e., 15 and 125 µM), to (i) permeabilize the outer membrane; (ii) both the outer and the inner membranes; and (iii) depolarize the inner membrane of Gram‐negative bacteria, including the *En. cloacae* DSM 129 strain (MIC_90_**NV716**_ = 20 µg/ml)^39^. The results obtained suggest that NV716 is capable of severely disrupting the integrity of the outer membrane of all Gram‐negative pathogens, with only a poor impact on the permeabilization of the inner membrane, as evidenced by low levels of ATP efflux^39^. In order to investigate more in depth the discrepancy observed with the potentiation of OX and CyC only on *En. cloacae* ATCC 23355, we decided to perform the same real‐time assays, but with increasing concentrations of NV716 (Figure S3), to evaluate the dose–response effect of this enhancer. For the tests focused on membrane integrity, Polymyxin B was used as the positive control due to its well‐known pore‐forming abilities^42^.

The impact of NV716 on outer‐membrane permeability was first assessed by monitoring the absorbance at 490 nm of the hydrolysis product nitrocefin. This non‐permanent yellow‐colored cephalosporin is indeed degraded by periplasmic β‐lactamases when the outer membrane has been permeabilized^24^. In the presence of Polymyxin B, the permeabilization was found to increase rapidly up to a plateau value reached at 10–1000 µg/ml (i.e., 100% permeabilization) (Figure S3A). Conversely, a classic sigmoidal dose–response curve was obtained with NV716, leading to significant ~155% membrane permeabilization (*p*‐value < 0.0001) at the highest concentration used as compared to Polymyxin B (Figure S3A). Inner‐membrane permeabilization was assessed by measuring the fluorescent signal generated when propidium iodide (PI) intercalates into bacterial DNA after permeabilization of both the outer and inner membranes^43^. Similarly, Polymyxin B increased PI fluorescence drastically, with an optimum reached at 1000 µg/ml, whereas NV716 failed to permeate the inner membrane irrespective of the concentration used (Figure S3B).

Finally, the capacity of NV716 to depolarize *En. cloacae* ATCC 23355 inner membrane was evaluated using the DiSC_3_(5) membrane potential‐sensitive dye that accumulates on polarized membranes, causing fluorescence to quench, but that is released when the membrane is depolarized, restoring the fluorescence signal^44^. In both cases, Polymyxin B and NV716 were found to depolarize the inner membrane of *En. cloacae* ATCC 23355 in a dose‐dependent manner (Figure S3C). However, at concentrations above 20 µg/ml, NV716 induced a stronger release of DiSC_3_(5) than Polymyxin B (Figure S3C). To confirm that the phenotypes observed in the presence of NV716 were the result of its effect on *En. cloacae* ATCC 23355 membranes rather than cell lysis, the survival of this bacterium under the operating conditions used in these real‐time assay series was then checked. Regardless of the concentration of NV716 tested, no significant differences in CFU counts were achieved compared to the positive control (Figure S3D). Taken together, the results of this dose‐dependent mechanistic study conducted on *En. cloacae* ATCC 23355 confirmed a mode of action of NV716 similar to that previously reported on several other Gram‐negative bacteria^38^, ^39^, ^40^, ^45^, i.e., permeabilization of the outer membrane and depolarization of the inner membrane.

The fact that NV716 has been shown to efficiently inhibit active efflux pumps of *P. aeruginosa*, *E. coli*, and *K. pneumoniae* ^24^, ^40^, ^45^, prompted us to investigate its effect on the active efflux of the 1,2'‐dinaphtylamine (DiNA) fluorescent probe^46^, ^47^ in *En. cloacae* ATCC 23355. First, the resistance/nodulation/division (RND) superfamily of efflux pumps^48^ was blocked by the addition of carbonyl cyanide‐*m*‐chlorophenylhydrazone (CCCP), an inhibitor of the proton motive force (PMF), and the bacteria were loaded with DiNA before re‐energizing the RND pumps with glucose^24^. When NV716 was added before glucose addition, a reduction of the fluorescence linked to DiNA efflux was observed (Figure S3F). However, the fact that, despite our best efforts, *En. cloacae* ATCC 23355 was only able to efflux 25% of the preloaded DiNA after the addition of glucose indicates a very weak efflux activity of this strain, thus limiting the potential impact of NV716 (Figure S3E).

### Synthesis and antibacterial activity of the CyC_32yne_ and the iBP_yne_OX “click‐ready” probes

**Figure 3:**
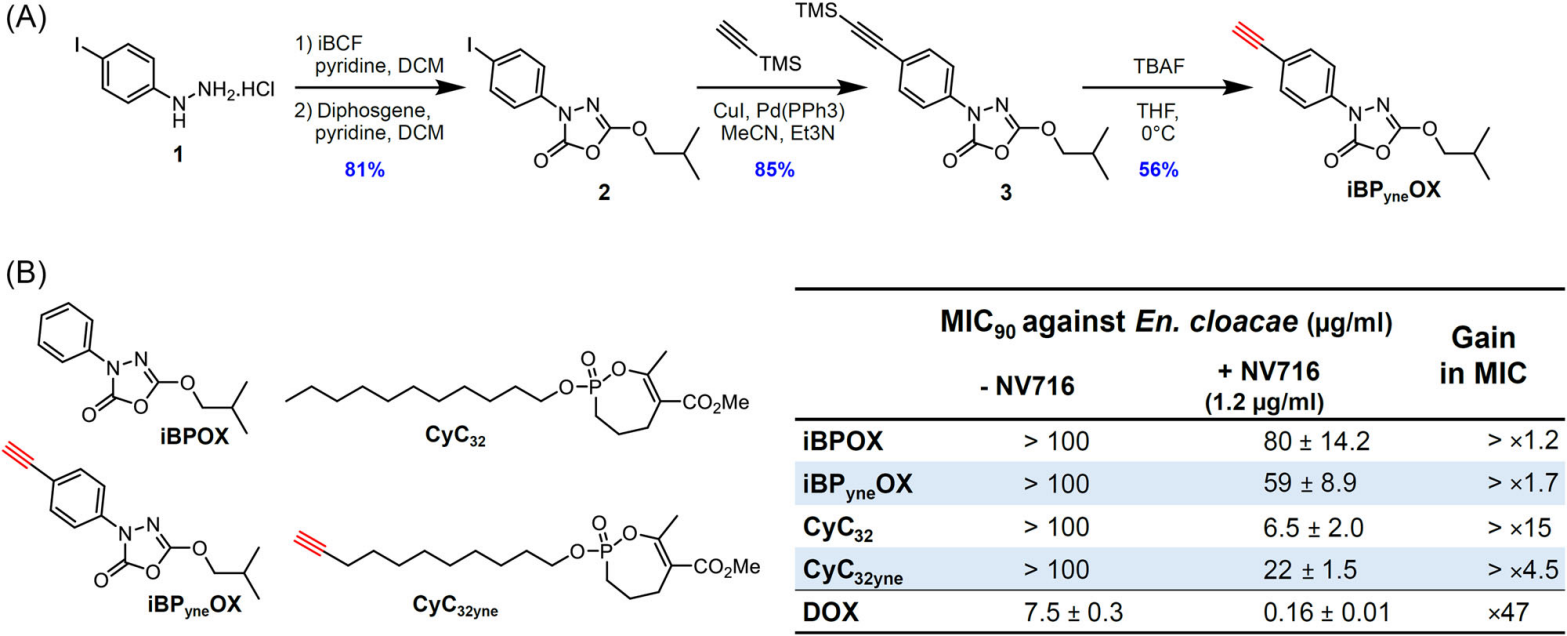
Synthesis and antibacterial activity of click‐ready activity‐based probes (ABPs). (A) Synthesis of the alkyne derivative of iBPOX bearing an alkyne group on the phenyl moiety, that is, iBP_yne_OX. (B) Structure of the two alkyne click‐ready probes used in this study, together with their parent molecules. The inset table shows the antibacterial activity of the four derivatives against *En. cloacae* ATCC 23355, reported as MIC_90_, that is, compound minimal concentration leading to 90% of growth inhibition as determined by the p‐iodonitrophenyltetrazolium violet (INT) assay following absorbance reading at 470 nm. Values are means of three independent assays performed in triplicate. DOX, doxycycline used as a control drug. INT, p‐iodonitrophenyltetrazolium violet.

We recently validated the use of new efficient CyC_yne_ activity‐based probes (ABPs), obtained by the direct introduction of a terminal alkyne function, as a means to capture the target proteins of these probes through direct CC‐ABPP^33^. Given the similar phenotypic mechanisms of action of NV716 in *En. cloacae* ATCC 23355 and *P. aeruginosa* PAO1, we decided to follow the same chemoproteomic CC‐ABPP approach to determine whether the specific antimicrobial activity observed against *En. cloacae* only was the consequence of unique and/or multiple targets in this Gram‐negative bacterium. To achieve this goal, click‐ready ABPs of the most potent inhibitors of *En. cloacae* ATCC 23355 growth, that is, iBP_yne_OX and CyC_32yne_ ^33^, respectively, were first synthesized (Figure 3).

The synthesis of CyC_32yne_ has been described in a previous study^33^. Regarding the alkyne derivative of iBPOX (i.e., iBP_yne_OX, Figure 3), we deliberately chose to position the alkyne function on the phenyl group rather than on the iso‐butyl motif, in order to avoid any steric hindrance near the reactive oxadiazolone core. The synthesis was first envisaged through the formation of the oxadiazolone ring using an alkyne phenyl‐hydrazine derivative. Unfortunately, we failed to synthesize the starting hydrazine from ethynyl‐aniline. The alkyne residue was thus introduced after the cyclization process^20^: the oxadiazolone ring was easily obtained starting from the commercial iodo‐phenylhydrazine **1** and isobutyl chloroformate (iBCF). Following the one‐pot (2 steps) cyclization process involving diphosgene, the corresponding iodo‐oxadiazolone **2** was used in Sonogashira coupling for alkyne introduction^49^. The final trimethylsilyl group deprotection of **3** was achieved using a catalytic amount of tetra‐n‐butylammonium fluoride (TBAF) to avoid ring opening^50^. This resulted in the synthesis of the desired iBP_yne_OX click‐ready ABP with a 39% overall yield (Figure 3A).

**Figure 4:**
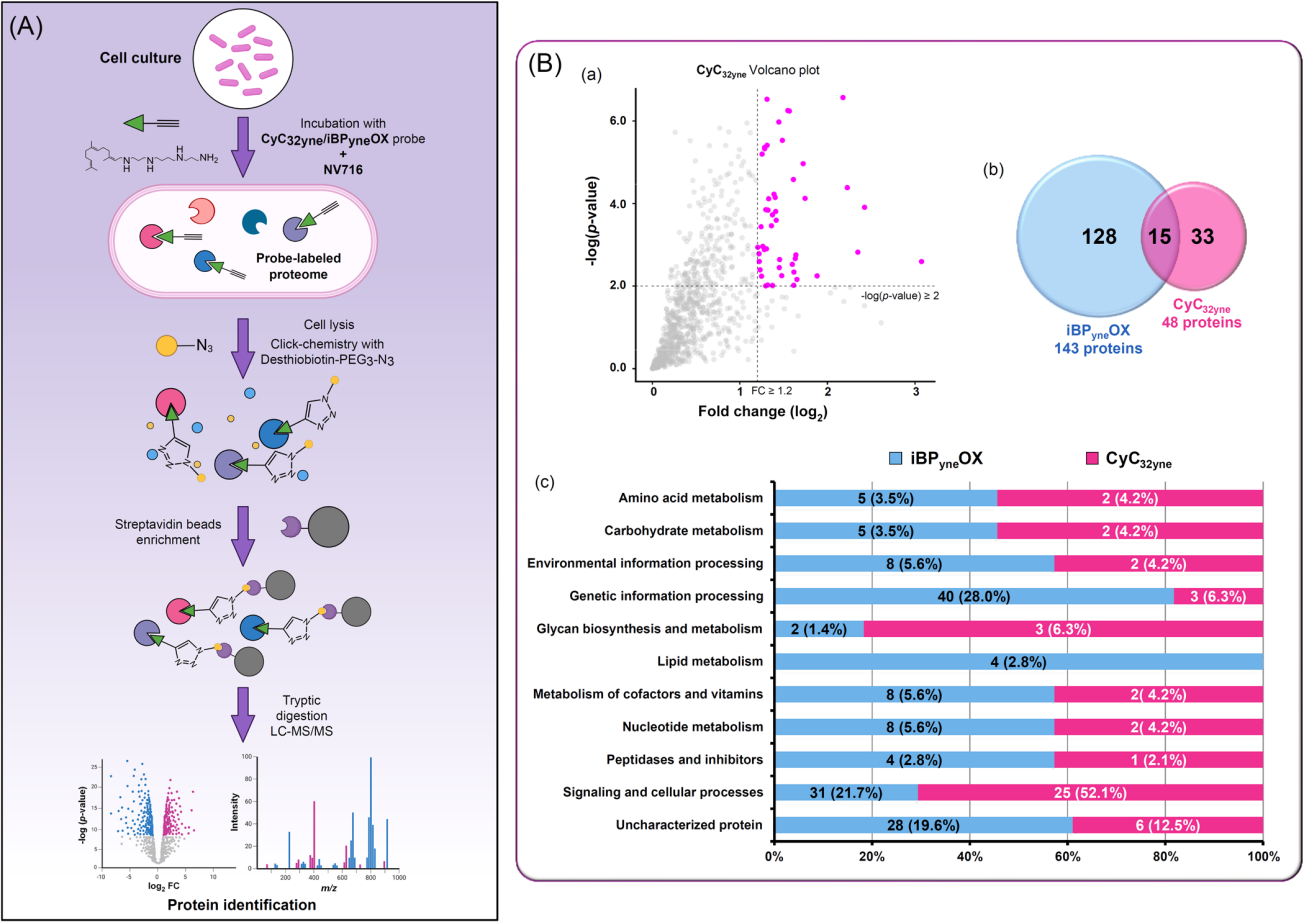
Click‐chemistry activity‐based profiling (CC‐ABPP) on *En. cloacae* ATCC 23355 culture. (A) CC‐ABPP typical workflow for the identification of proteins covalently bound to OX_yne_/CyC_yne_ inhibitors. *En. cloacae* ATCC 23355 culture was pretreated with either the iBP_yne_OX–NV716 or the CyC_32yne_–NV716 combination before cell lysis and click reaction with the Desthiobiotin‐PEG_3_‐N_3_ reporter. Samples were then treated with streptavidin–agarose beads for the capture and enrichment of labeled proteins, followed by tryptic digestion. Tandem mass spectrometry analyses and subsequent differential peptides analysis led to the identification of the OX and CyC target enzymes. Created in BioRender (https://BioRender.com/q94r710). (B) Proteomics analysis of CC‐ABPP applied to *En. cloacae* ATCC 23355 culture. (a) Volcano plot of the proteomics analysis of CyC_32yne_ showing the significance of the two‐sample *t*‐test (‐log_10_(*p*‐value)) vs. fold change (log_2_(LFQ normalized intensity in CyC_32yne_ vs. nonspecific conditions)) on the *y* and *x* axes, respectively. Only the proteins with a positive fold‐change are plotted here. The dashed lines indicate the threshold of *p*‐value ≤ 0.01 and a fold change ≥ 1.2. (b) Venn diagram showing the total number of proteins differentially captured with iBP_yne_OX or CyC_32yne_ at a *p*‐value ≤ 0.01 and a fold change (log_2_) ≥ 1.2. (c) Functional categories of *En. cloacae* ATCC 23355 proteins identified following treatment with the iBP_yne_OX–NV716 or CyC_32yne_–NV716 combination according to the functional classification system of the KEGG database^51^, ^52^. The numbers correspond to the total number of proteins identified in each category, with the corresponding percentage in parentheses based on either the 143 or 48 proteins identified with iBP_yne_OX or CyC_32yne_, respectively. Proteins without annotation are denoted as uncharacterized proteins.

The antimicrobial potency of the two alkyne derivatives in the presence or absence of NV716 was evaluated against *En. cloacae* ATCC 23355 and compared with that of their respective parent molecules (Figure 3B). Although no antimicrobial activity was observed in the absence of NV716, comparable antibacterial activities against *En. cloacae* ATCC 23355 were obtained in the presence of NV716 for each inhibitor/probe pair, that is, iBPOX/iBP_yne_OX (80/59 µg/ml) and CyC_32_/CyC_32yne_ (6.5/22 µg/ml), respectively (Figure 3B). Following this prerequisite, iBP_yne_OX and CyC_32yne_ were used as affinity‐based probes in a CC‐ABPP strategy (Figure 4A).

### *En. cloacae* ATCC 23355 target proteins identified by CC‐ABPP using the CyC_32yne_ and iBP_yne_OX affinity‐based probes

To take into account the critical issue of penetration of our probes inside *En. cloacae* ATCC 23355, each OX_yne_ and CyC_yne_ molecule was incubated with a bacterial culture of this Gram‐negative bacterium in the presence of NV716. Accordingly, mid‐log‐phase *En. cloacae* ATCC 23355 cells were incubated with each inhibitor/enhancer pair: iBP_yne_OX–NV716 or CyC_32yne_–NV716 (or DMSO‐NV716 as a control for the nonspecific condition). After cell lysis, each total lysate was subjected to a click‐chemistry reaction using the copper(I)‐catalyzed Huisgen's 1,3‐dipolar cycloaddition reaction^53^ to form a triazole cycle between the probe–enzyme complex and the Desthiobiotin–PEG_3_–N_3_ reporter. The samples were enriched with streptavidin–agarose beads, then tryptic‐digested, and the obtained peptides were analyzed by liquid chromatography‐tandem mass spectrometry (LC–MS/MS), followed by label‐free quantification analysis (Figure 4A). Comparative analysis between the proteomes of the DMSO control sample and each of the OX_yne_ **‐** & CyC_yne_‐treated samples enabled a volcano plot representation (Figure 4B‐a). Only proteins identified after applying *p*‐value ≤ 0.01 and log_2_(fold change) ≥ 1.2 thresholds on the proteomics analysis results were selected, therefore leading to a panel of 143 and 48 enzyme candidates for the iBP_yne_OX–NV716 and the CyC_32yne_–NV716 combinations, respectively (Figure 4B, Tables S3 and S4).

As shown in the Venn diagram (Figure 4B‐b), and despite their closely related mechanism of action^30^, both inhibitor probes share only 15 common targets (Table S5). It is noteworthy that, as depicted in the functional categorization profile of the captured proteins (Figure 4B‐c), similar biological processes were, however, targeted by the two inhibitors. This result reflects the complementary selectivity of the two reactive warheads, that is, the oxadiazolone ring and the enolphosphonate cycle, which in fact differ in their electrophilicity and enzyme specificity, as already observed in previous studies^20^, ^22^, ^31^, ^32^. With the iBP_yne_OX, a significant number of ribosomal proteins involved in “Genetic information processing” were observed, suggesting stronger and enhanced activity in protein synthesis following high stress treatment with this OX–NV716 association. Such a feature is also observed for the proteins belonging to the “Signaling and cellular processes” category, which represent 21.7% and 52.1% of the total proteins captured with iBP_yne_OX and CyC_32yne_ in bacterial culture, respectively. Notably, numerous transporters and stress response proteins were found, such as the Phage shock protein C (ECL_01778), which is produced when bacterial membranes are damaged.

To further analyze the results of these CC‐ABPP experiments and compare them with previous work on the capture of target enzymes in mycobacteria^30^, ^33^, the corresponding orthologs in *M. tuberculosis* H37Rv and/or *M. abscessus* ATCC 19977 were also shown using the KEGG database^51^, ^52^ and then cross‐referenced with the OrtholugeDB online database^54^ (Table S5). Interestingly, 125 out of the 176 proteins identified from *En. cloacae* ATCC 23355 culture have orthologs in the *M. tuberculosis* and/or *M. abscessus* genome. Of these, 63 proteins had already been identified as potential targets of the OX/CyC probes through ABPP on culture and/or total lysate of *M. abscessus* ^22^, ^31^ and *M. tuberculosis* ^20^, ^32^, ^33^ (Tables 3 and S5), 5 of them being annotated as essential genes according to the essential genes database (DEG)^55^. These included three ribosomal proteins (ECL_04612/MAB_3752c; ECL_04692/Rv0706/MAB_3815c; ECL_04698/Rv0700/MAB_3821c), the cell division protein FtsZ (ECL_00892/Rv2150c/MAB_2009), and the 3‐oxoacyl‐[acyl‐carrier‐protein] reductase FabG (ECL_02543/Rv1483/MAB_2723c), which are highly conserved both in mycobacteria and in Gram‐negative ESKAPEE bacteria. In terms of subcellular localization, the fact that these target proteins originate from the cell envelope and the cytoplasm of this Gram‐negative bacterium is consistent with all our previous studies, where identified protein targets of the OX and CyC in *M. tuberculosis* ^20^, ^32^, ^33^ and *M. abscessus* ^22^, ^31^ were localized in both cellular compartments. Taken together, these findings clearly support the proof of concept that the polyamino‐isoprenyl enhancer NV716 has the ability to render the OX and CyC inhibitors active against *En. cloacae* ATCC 23355, while allowing them to retain their multi‐target properties.

**Table 3:** *En. cloacae* ATCC 23355 target proteins identified by CC‐ABPP using the iBP_yne_OX and CyC_32yne_ affinity‐based probes, together with their respective *Mycobacterium tuberculosis* and/or *Mycobacterium abscessus* orthologs^a^.

			Ortholog			
iBP_yne_OX	CyC_32yne_	Gene name	*M. tuberculosis*	*M. abscessus*	Protein name	Pathway
+		ECL_00157	Rv2335	MAB_1148c^b^	Serine acetyltransferase	Amino acid metabolism
+		ECL_01853	Rv2858c^b^	MAB_4203^b^	Gamma‐aminobutyraldehyde dehydrogenase	Amino acid metabolism
+		ECL_03989	Rv3565^b^	MAB_3582	GntR family transcriptional regulator	Amino acid metabolism
+		ECL_05036	Rv1908c^b^	MAB_2470c^b^	Catalase‐peroxidase	Amino acid metabolism
	+	ECL_03341	Rv1600	MAB_2669c^b^	Histidinol‐phosphate aminotransferase	Amino acid metabolism
	+	ECL_05014	Rv3003c^b^	MAB_3323c^b^	Acetolactate synthase	Amino acid metabolism
	+	ECL_04055	Rv0533c^b^	MAB_3192c	Cryptic 6‐phospho‐beta‐glucosidase	Carbohydrate metabolism
+		ECL_01284	Rv0844c^b^	MAB_4519c^b^	Two‐component system, NarL family, response regulator, fimbrial Z	Environmental information processing
+		ECL_01619	Rv0844c^b^	MAB_0060^b^	Nitrate/nitrite response regulator	Environmental information processing
+		ECL_04457	Rv3565^b^	MAB_3387^b^	Methyl‐accepting chemotaxis sensory transducer	Environmental information processing
+	+	ECL_01778	Rv2027c^b^	MAB_3715c	Phage shock protein C	Genetic information processing
+		ECL_00317	Rv0054^b^	MAB_4898c	Single‐stranded DNA‐binding protein	Genetic information processing
+		ECL_00242	Rv0641^b^	MAB_3892c	Large ribosomal subunit protein uL1	Genetic information processing
+		ECL_00597	Rv0056^b^	MAB_4896c	Large ribosomal subunit protein bL9	Genetic information processing
+		ECL_00594	Rv0053^b^	MAB_4899c	Small ribosomal subunit protein bS6	Genetic information processing
+		ECL_00109	Rv1644	MAB_0572^b^	tRNA (guanosine(18)‐2'‐O)‐methyltransferase	Genetic information processing
+		ECL_00240	Rv0639^b^	MAB_3894c^b^	Transcription termination/antitermination protein NusG	Genetic information processing
+		ECL_03489	Rv2534c	MAB_2837c^b^	Elongation factor P‐like protein	Genetic information processing
+		ECL_04612	Rv3443c	MAB_3752c^b^	Large ribosomal subunit protein uL13	Genetic information processing
+		ECL_04698	Rv0700^b^	MAB_3821c^b^	Small ribosomal subunit protein uS10	Genetic information processing
+		ECL_05119	Rv1629*	MAB_2615c	DNA polymerase I	Genetic information processing
+		ECL_04695	Rv0703	MAB_3818c^b^	Large ribosomal subunit protein uL23	Genetic information processing
+		ECL_04706	Rv0683	MAB_3850c^b^	Small ribosomal subunit protein uS7	Genetic information processing
+		ECL_04696	Rv0702^b^	MAB_3819c^b^	Large ribosomal subunit protein uL4	Genetic information processing
+		ECL_04611	Rv3442c^b^	MAB_3751c	Small ribosomal subunit protein uS9	Genetic information processing
+		ECL_04692	Rv0706	MAB_3815c^b^	Large ribosomal subunit protein uL22	Genetic information processing
+		ECL_04697	Rv0701	MAB_3820c^b^	Large ribosomal subunit protein uL3	Genetic information processing
+		ECL_03742	Rv2587c^b^	MAB_0803	Cell division protein ZipA	Genetic information processing
+		ECL_01194	Rv2462c^b^	MAB_1580^b^	Trigger factor Tig	Genetic information processing
+	+	ECL_02592	Rv0074^b^	MAB_3236c	Glucans biosynthesis glucosyltransferase H	Glycan biosynthesis and metabolism
	+	ECL_04529	Rv2587c^b^	MAB_0108c	Penicillin‐binding protein activator LpoA	Glycan biosynthesis and metabolism
+		ECL_02543	Rv1483^b^	MAB_2723c^b^	3‐oxoacyl‐[acyl‐carrier‐protein] reductase FabG	Lipid metabolism
+		ECL_03683	Rv0243^b^	MAB_1002^b^	3‐ketoacyl‐CoA thiolase	Lipid metabolism
+		ECL_01173	Rv1416^b^	MAB_2795c	6,7‐dimethyl‐8‐ribityllumazine synthase	Metabolism of cofactors and vitamins
+		ECL_02952	Rv0984^b^	MAB_1079	Molybdenum cofactor biosynthesis protein B	Metabolism of cofactors and vitamins
+		ECL_00113	Rv1389	MAB_2823c^b^	Guanylate kinase	Nucleotide metabolism
+		ECL_02171	Rv0484c	MAB_4060^b^	NADP‐dependent 3‐hydroxy acid dehydrogenase YdfG	Nucleotide metabolism
+		ECL_02486	Rv1699^b^	MAB_2364	CTP synthase (glutamine hydrolyzing)	Nucleotide metabolism
+		ECL_02744	Rv1712^b^	MAB_2371*	Cytidylate kinase (CK)	Nucleotide metabolism
+		ECL_03774	Rv0780	MAB_0689^b^	Phosphoribosylaminoimidazole‐succinocarboxamide synthase	Nucleotide metabolism
+		ECL_03860	Rv2445c^b^	MAB_1606^b^	Nucleoside diphosphate kinase	Nucleotide metabolism
+		ECL_00721	Rv2461c^b^	MAB_1581*	ATP‐dependent Clp protease proteolytic subunit	Peptidases and inhibitors
+		ECL_04557	Rv3610c	MAB_0533^b^	ATP‐dependent zinc metalloprotease FtsH	Peptidases and inhibitors
+		ECL_05045	Rv2110c^b^	MAB_2172	ATP‐dependent protease subunit HslV	Peptidases and inhibitors
	**+**	ECL_02464	Rv0724^b^	MAB_3789c	Protease 4	Peptidases and inhibitors
+		ECL_02504	Rv1027c	MAB_3250c^b^	DNA‐binding transcriptional regulator PhoP	Signaling and cellular processes
+		ECL_00892	Rv2150c^b^	MAB_2009	Cell division protein FtsZ	Signaling and cellular processes
+		ECL_01160	Rv2428^b^	MAB_4408c*	Alkyl hydroperoxide reductase C	Signaling and cellular processes
+		ECL_03521	Rv0844c^b^	MAB_1522^b^	Transcriptional regulatory protein RcsB	Signaling and cellular processes
+		ECL_03943	Rv2916c	MAB_3237c^b^	Signal recognition particle protein	Signaling and cellular processes
	+	ECL_00368	Rv1200	MAB_2263c^b^	Proline/glycine betaine transporter	Signaling and cellular processes
	+	ECL_01828	Rv1239c^b^	MAB_4693	Zinc transport protein ZntB	Signaling and cellular processes
	+	ECL_03532	Rv3476c	MAB_2263c^b^	sn‐glycerol‐3‐phosphate transporter	Signaling and cellular processes
	+	ECL_04977	Rv1239c^b^	MAB_1378c	Magnesium transport protein CorA	Signaling and cellular processes
+		ECL_00740	Rv0234c^b^	MAB_4322	Putative aldehyde dehydrogenase	Uncharacterized protein
+		ECL_01005	Rv2971^b^	MAB_1528c	NADP‐dependent oxidoreductase domain‐containing protein	Uncharacterized protein
+		ECL_03029	Rv0045c^b^	MAB_1169	AB hydrolase‐1 domain‐containing protein	Uncharacterized protein
+		ECL_02982	Rv0489^b^	MAB_4049c	2,3‐bisphosphoglycerate‐dependent phosphoglycerate mutase	Uncharacterized protein
+		ECL_04029	Rv3400^b^	MAB_2130	Fructose‐1‐phosphatase	Uncharacterized protein
+		ECL_04104	Rv2392^b^	MAB_1661c	Phosphoadenosine 5'‐phosphosulfate reductase	Uncharacterized protein
+		ECL_03668	Rv3708c^b^	MAB_0344	Aspartate‐semialdehyde dehydrogenase	Uncharacterized protein
+		ECL_03953	Rv3100c	MAB_3473c^b^	SsrA‐binding protein	Uncharacterized protein
+		ECL_04636	Rv0149^b^	MAB_4603c^b^	Alcohol dehydrogenase	Uncharacterized protein
	+	ECL_00728	Rv0223c^b^	MAB_4484^b^	Aldehyde dehydrogenase	Uncharacterized protein

^a^ *En. cloacae* ATCC 23355 orthologs were retrieved using the KEGG database^51^, ^52^ and then cross‐referenced with the OrtholugeDB online database^54^. ^b^ Proteins previously identified from ABPP experiments on culture or total lysates of *M. abscessus* ^22^, ^31^ or *M. tuberculosis* ^20^, ^32^, ^33^ with the OX and CyC.

### Identification of potential target proteins from a total lysate of *P. aeruginosa* PAO1 by CC‐ABPP using the CyC_32yne_ affinity‐based probe

In a previous work, we have shown that the CyC_32‐Dansyl_ fluorescent probe was able to efficiently label some bacterial enzymes present in a total lysate of *P. aeruginosa* PAO1^35^. In this context, we decided to apply the same CC‐ABPP approach on a CyC_32yne_‐treated total lysate of this Gram‐negative bacterium. Following proteomic analysis, 36 potential target enzymes have been identified, of which 10 are annotated as essential proteins for bacterial growth^55^ (Table S6). Interestingly, 30 of these 36 targets have *En. cloacae* genomic orthologs (Table S6), including 5 proteins that have also been captured from the *En. cloacae* ATCC 23355 culture (Table 4). These included the nucleoside diphosphate kinase Ndk (PA3807/ECL_03860/Rv2445c/MAB_1606) involved in Nucleotide metabolism; the outer membrane LPS‐assembly protein LptD (PA0595/ECL_00852) involved in the assembly of LPS; the essential Trigger factor Tig (PA1800/ECL_01194/Rv2462c/MAB_1580), which functions as a peptidyl‐prolyl *cis–trans* isomerase and is involved in the genetic information processing pathway; and finally, an acyl‐CoA thioesterase (PA2856/ECL_01261) and the essential 3‐oxoacyl‐[acyl‐carrier‐protein] reductase FabG (PA2967/ECL_02543/Rv1483/MAB_2723c), both involved in the lipid metabolism pathway (Table 4). Notably, Rv2445c/MAB_1606, Rv2462c/MAB_1580, and Rv1483/MAB_2723c are part of the set of previously identified target proteins of the OX/CyC from *M. tuberculosis* ^20^, ^32^, ^33^ and *M. abscessus* ^22^, ^31^. In particular, based on the fact that the latter five proteins, including two essential enzymes, are highly conserved in all Gram‐negative ESKAPEE bacteria, we can hypothesize that the OX and CyC inhibitors should be able to block the growth of these pathogens, provided that they penetrate and enter the bacteria, as in the case of *En. cloacae*.

**Table 4:** CyC_32yne_ target proteins identified in *Pseudomonas aeruginosa* PAO1 as well as in *En. cloacae* ATCC 23355 culture by LC‐ESI‐MS/MS analysis^a^.

		*En. cloacae* ortholog				
Gene name	Essentiality	Gene name	Sequence identity	Overlap	Protein name	Pathway
PA1800	Essential	ECL_01194	0.507	426	Trigger factor Tig^b^	Genetic information processing
PA2967	Essential	ECL_02543	0.642	246	3‐oxoacyl‐[acyl‐carrier‐protein] reductase FabG^b^	Lipid metabolism
PA2856	Non‐Essential	ECL_01261	0.477	193	acyl‐CoA thioesterase	Lipid metabolism
PA3807	Non‐Essential	ECL_03860	0.629	143	Nucleoside diphosphate kinase Ndk^b^	Nucleotide metabolism
PA0595	Non‐Essential	ECL_00852	0.306	797	Lipopolysaccharide‐assembly protein LptD	Signaling and cellular processes

^a^ *En. cloacae* ATCC 23355 orthologs were retrieved using the KEGG database^51^, ^52^ and then cross‐referenced with the OrtholugeDB online database^54^. The essentiality of each *P. aeruginosa* PAO1 gene was checked using the essential genes database (DEG)^55^. ^b^Proteins previously identified from ABPP experiments on culture or total lysates of *Mycobacterium abscessus* ^22^, ^31^ or *Mycobacterium tuberculosis* ^20^, ^32^, ^33^ with OX and CyC.

## DISCUSSION

As the race for efficient, broad‐spectrum antibiotics is slowing down, new strategies to eradicate drug‐resistant bacteria are thus needed. In this context, “adjuvant therapy” represents a promising but still underexplored area. Antibiotic adjuvants are molecules with little or no antibacterial activity that can enhance the activity of existing antibiotics by minimizing, bypassing, or directly blocking the resistance mechanisms associated with them^12^. The two main classes of adjuvants currently in development are β‐lactamase inhibitors, the best example of which is the clinically approved and widely prescribed Augmentin™, a combination of the β‐lactam antibiotic amoxicillin and the β‐lactam‐based inhibitor clavulanic acid^56^, and membrane disruptors^15^.

Membrane‐targeting compounds usually bypass passive resistance mechanisms such as the permeability barrier of Gram‐negative bacteria and can also block efflux mechanisms^12^. Among the reported membrane‐disrupting adjuvants, pentamidine, an antiprotozoal drug, was able to potentiate novobiocin against colistin‐resistant strains of *A. baumannii*, and interestingly, a combination of pentamidine (10 mg/kg) and novobiocin (50 mg/kg) significantly reduced splenic bacterial load in an in vivo model of infected mice^57^. Recently, P35, a new synthetic analog of pentamidine, demonstrated increased in vivo efficacy and reduced toxicity in a mouse model of *A. baumannii* infection when combined with novobiocin^58^. Similar extensive stucture activity relationship (SAR) studies conducted on polymyxin B‐derived molecules led to the identification of SPR741^59^. This molecule, which showed synergistic effects with various antibiotics, notably by significantly reducing bacterial burden and promoting animal survival in a murine pulmonary model of *A. baumannii* infection, has completed Phase I clinical trials^59^, ^60^, ^61^. Apart from these successful examples, few membrane‐disrupting adjuvants have been the subject of in vivo studies^15^.

In this challenging context, here, we have tested the association of multi‐target inhibitors of (Ser/Cys)‐based enzymes, namely, OX and CyC, with a known antibiotic adjuvant: the polyamino‐isoprenyl derivative NV716. Indeed, although OX and CyC displayed promising antibacterial activity against pathogenic mycobacterial species, such as *M. abscessus* and *M. tuberculosis*, with no toxicity to mammalian host cells, they are inactive against Gram‐negative bacteria due to their incapacity to cross their outer membrane. In this study, we showed that NV716, which has been shown to efficiently potentiate disused antibiotics against Gram‐negative bacteria, such as *P. aeruginosa*, *E. coli*, or *K. pneumoniae* ^24^, ^26^, ^40^, ^45^, was able to sensitize the ESKAPEE bacterium *En. cloacae* ATCC 23355 to some OX and CyC compounds. Mechanistically, we confirmed that the NV716 potentiation likely occurs through effective strategies to fight Gram‐negative bacteria^62^, ^63^: permeabilization of the outer membrane and depolarization of the inner membrane of *En. cloacae* ATCC 23355.

However, the MIC_90_ of NV716 against the five clinical isolates of *En. cloacae* was found to be 16 times higher than that of the above reference strain. This finding suggests that the ability of this enhancer to potentiate the OX and CyC compounds may strongly depend on the isolate resistance and efflux phenotypes (Table S1)^41^, as shown with reference drugs (Table S2). Moreover, while the iBPOX–NV716 association showed only poor antibacterial activity against the five clinical strains, the CyC_32_–NV716 combination was able to efficiently inhibit the growth of *Ecl71* and *Ecl93* (Table 2). *Ecl71* was characterized as a wild‐type clinical strain, with a basal efflux profile. *Ecl53* and *Ecl136* are highly resistant isolates, with the same substitutions in AcrA and AcrR efflux pump components. Strains *Ecl80* and *Ecl93* showed the same substitutions in the efflux key gene *tolC*, but only strain *Ecl80* showed mutations in the multidrug efflux pump component OqxA, known to confer resistance to multiple agents including fluoroquinolones^64^. Since NV716 has nearly no effect on the efflux in *En. cloacae* ATCC 23355 (Figure S3), we hypothesize that the observed resistance of *Ecl53*, *Ecl136*, and *Ecl80* to iBPOX and CyC_32_ in the presence of NV716 may result from the overexpressing efflux phenotypes of these three isolates, thereby preventing the accumulation of the two inhibitors within the bacteria. This hypothesis is supported by evidence that NV716, even at a concentration as low as 0.015× MIC_90_*Ecl*‐isolate_, enhances the antibacterial activity of doxycycline against these three clinical isolates (Table S2) by permeabilizing their outer membrane.

However, the inability of NV716 to potentiate the OX and CyC antibacterial activity on Gram‐negative ESKAPEE bacteria other than *En. cloacae* ATCC 23355 and some clinical isolates despite a similar mechanism of action^40^, ^45^ suggests that the permeation of the outer membrane caused by NV716 may not be sufficient to allow the penetration, accumulation, and subsequent antibacterial activity of the OX and CyC compounds.

The hydrophobic/hydrophilic balance of a drug candidate is indeed a critical issue that determines its physicochemical compatibility with the bacterial outer membrane and thus its ability to penetrate the bacteria. In this context, the distribution coefficient, LogD, is a widely used metric of the lipophilicity for a given molecule. Here, the calculated cLogD at pH 7.4 of our OX and CyC compounds using Marvin Suite (ChemAxon) shows that they all display a high lipophilic nature (2 < cLogD < 6). This property is in line with their very good penetration and antibacterial activity on mycobacterial species, which have a very complex, hydrophobic, and thick lipid‐rich membrane consisting of up to 60% cell wall lipids^65^, ^66^. In comparison, the cell wall of Gram‐negative bacteria contains no more than 20% of lipid content. A recent study conducted on cycline derivatives showed that their potentiation by NV716 against *P. aeruginosa* correlated with their hydrophobic/hydrophilic balance^18^, the most active derivatives having a cLogD < –1.8 (pH 7.4), indicative of very high hydrophilicity. Regarding the OX and CyC compounds, their high hydrophobicity might limit their compatibility with the Gram‐negative outer membrane. Indeed, it is well acknowledged that variations in the chemical structure of LPS, for example, in the composition of the sugar head group, can strongly influence the activity of membrane‐active compounds and so their antimicrobial activity^10^, ^67^. Taken together, all these findings may provide some insights into the differential potentiation of OX and CyC compounds observed among the four bacterial species tested. This latter point is of particular interest, considering that the CC‐ABPP approach on bacterial culture (Figure 4) revealed that OX and CyC compounds still retain their multi‐target properties on Gram‐negative bacteria (Tables 3, 4 and S3–S6). In addition, these proteomic data also indicate that the presence of NV716, which is required for the activity of the compounds, strongly alters the bacterial metabolism and may influence the nature of the proteins captured by the OX and CyC probes. Furthermore, the fact that several highly conserved proteins across all Gram‐negative ESKAPEE bacteria (70‐100% positive hits, at least 60% identities when BLASTed) have been identified is very encouraging and should provide good insights into the antibacterial potential of our OX and CyC compounds against bacterial pathogens.

In summary, this study points out the efficiency of NV716 as an antibiotic enhancer that enables the OX and CyC multi‐target inhibitors to impair the bacterial growth of the Gram‐negative *En. cloacae* ATCC 23355 bacterium. Furthermore, from all our data, we anticipate that the synthesis of novel OX and CyC inhibitors with enhanced hydrophilicity (i.e., negative cLogD) should facilitate their penetration, maximize the physicochemical compatibility, and thus boost their bactericidal activity against other clinically relevant Gram‐negative ESKAPEE species. Such studies are currently ongoing and will be reported in due course.

## MATERIALS AND METHODS

All detailed protocols are provided in the Supporting Information.

### Chemistry

#### Synthesis of OX and CyC activity‐based probes

The Cyclipostins analogs CyC_32_, CyC_32yne_ and CyC_32‐Dansyl_, as well as the oxadiazolone derivative HPOX, were synthesized as described previously^20^, ^33^, ^35^. Stock solutions (10 mM) of the OX and CyC molecules (purity of ≥95%) were prepared in dimethyl sulfoxide (DMSO) and stored at 4°C. See Figure S4 for NMR spectra of the new compounds synthesized to access the iBP_yne_OX probe and for additional details.

***3‐(4‐iodophenyl)‐5‐isobutoxy‐1,3,4‐oxadiazol‐2(3H)‐one (2)***. A solution of *p*‐iodophenylhydrazine hydrochloride (2.3 g, 8.50 mmol, 1 equiv.) in dry pyridine (18 ml) was stirred at room temperature for 15 min. The mixture was then cooled to 0°C and a solution of isobutyl chloroformate (1.12 ml, 8.50 mmol, 1 equiv.) in dry CH_2_Cl_2_ (6 ml) was slowly added. The mixture was stirred at 0°C for 30 min, followed by 1 h at room temperature. The mixture was then diluted with dry CH_2_Cl_2_ (36 ml) and dry pyridine (9 ml) and cooled to 0°C. A solution of diphosgene (1.03 ml, 8.50 mmol, 1 equiv.) in dry CH_2_Cl_2_ (12 ml) was added dropwise. The mixture was stirred at 0°C for 30 min, followed by 2 h at room temperature. The mixture was then degassed with N_2_ to eliminate the remaining phosgene, treated with 1 M HCl (60 ml), and extracted with Et_2_O (2 × 100 ml). Organic layers were washed with a Na_2_S_2_O_3_ 10% solution (60 ml) and brine, dried over MgSO_4_, and solvents were removed under reduced pressure. The crude product was then adsorbed on silica and purified through silica gel chromatography with a gradient pentane/Et_2_O 100/0 to 95/5 to yield oxadiazolone **2** (2.49 g, 7.50 mmol, 81% over 2 steps) as a yellow solid. Analytical data for (**2**): R_*f*_ (cyclohexane/EtOAc 90:10, *v/v*) 0.55; ^1^H NMR (300 MHz, CDCl_3_): δ 7.71 (d,^3^ *J*_H,H_ = 9.0 Hz, 2H, *H*_Ar_), 7.57 (d,^3^ *J*_H,H_ = 9.0 Hz, 2H, *H*_Ar_), 4.16 (d,^3^ *J*_H,H_ = 6.6 Hz, 2H, C*H*_2_‐O), 2.15 (non,^3^ *J*_H,H_ = 6.7 Hz, 1H, C*H*‐(CH_3_)_2_), 1.04 (d,^3^ *J*_H,H_ = 6.7 Hz, 6H, CH‐(C*H*_*3*_ *)*_*2*_); ^13^C NMR (75 MHz, CDCl_3_): δ 155.6, 148.2, 138.2 (2C), 136.2, 119.7 (2C), 89.4, 77.7, 27.9, 18.8 (2C).

***5‐isobutoxy‐3‐(4‐((trimethylsilyl)ethynyl)phenyl)‐1,3,4‐oxadiazol‐2(3H)‐one (3).*** A solution of **2** (1 g, 2.99 mmol, 1.0 equiv.), trimethylsilylacetylene (634 µl, 4.49 mmol, 1.5 equiv.), and dry distilled Et_3_N (1.04 ml, 7.48 mmol, 2.5 equiv.) in dry MeCN (10 ml) was cooled to 0°C. Then, Pd(PPh_3_)_4_ (243 mg, 0.21 mmol, 0.07 equiv.) and CuI (399 mg, 2.09 mmol, 0.7 equiv.) were added and the mixture was stirred at 0°C for 30 min, followed by 3 h at room temperature. The mixture was then filtered on Celite® and the solvent was removed under reduced pressure. The crude was then adsorbed on silica and purified through silica gel chromatography with a gradient pentane/Et_2_O 100/0 to 95/5 to yield oxadiazolone **3** (840 mg, 2.54 mmol, 85%) as an off‐white solid. Analytical data for (**3**): R_*f*_ (cyclohexane/EtOAc 90:10 *v/v*) 0.55; ^1^H NMR (300 MHz, CDCl_3_): δ 7.75 (d,^3^ *J*_H,H_ = 9.0 Hz, 2H, *H*_Ar_), 7.50 (d,^3^ *J*_H,H_ = 9.0 Hz, 2H, *H*_Ar_), 4.17 (d,^3^ *J*_H,H_ = 6.6 Hz, 2H¸ C*H*_2_‐O), 2.15 (non,^3^ *J*_H,H_ = 6.7 Hz, 1H, C*H*‐(CH_3_)_2_), 1.04 (d,^3^ *J*_H,H_ = 6.8 Hz, 6H, CH‐(C*H*_*3*_ *)*_*2*_), 0.25 (s, 9H, (C*H*_*3*_)_*3*_‐Si); ^13^C NMR (75 MHz, CDCl_3_): *δ* 155.6, 148.2, 136.2, 133.0 (2C), 120.2, 117.5 (2C), 104.5, 94.7, 77.7, 27.9, 18.8 (2C), 0.1 (3C).

***3‐(4‐ethynylphenyl)‐5‐isobutoxy‐1,3,4‐oxadiazol‐2(3H)‐one or iBP***_***yne***_ ***OX.*** A solution of **3** (828 mg, 2.51 mmol, 1 equiv.) in dry THF (25 ml) was cooled to 0°C. A solution of TBAF 1M (500 µl, 0.50 mmol, 0.20 equiv.) was slowly added. The mixture was stirred at 0°C for 2 h and was then quenched with a NaHCO_3_ saturated solution (50 ml). The resulting solution was extracted with Et_2_O (2 × 50 ml), organic layers were gathered, washed with brine, dried over MgSO_4_, and solvents were evaporated under reduced pressure. The crude was then adsorbed on silica and purified through silica gel chromatography with a gradient pentane/Et_2_O 100/0 to 90/10. The product was then further purified by recrystallization in hot pentane to yield the desired iBP_yne_OX probe (360 mg, 1.39 mmol, 56%) as a yellow solid. Analytical data for iBP_yne_OX: R_*f*_ (cyclohexane/EtOAc 90:10, *v/v*) 0.27; Mp 71–72°C; IR (neat) *ν* 3301 (C≡C–H), 2968, 2878 (C–H), 1779 (C═O), 1606, 1509, 1470 (C═C) cm^−1^; ^1^H NMR (300 MHz, CDCl_3_): *δ* 7.78 (d,^3^ *J*_H,H_ = 8.9 Hz, 2H, *H*_Ar_), 7.53 (d,^3^ *J*_H,H_ = 8.9 Hz, 2H, *H*_Ar_), 4.17 (d,^3^ *J*_H,H_ = 6.6 Hz, 2H, C*H*_2_‐O), 3.09 (s, 1H, C≡C*H*), 2.17 (non,^3^ *J*_H,H_ = 6.7 Hz, 1H, C*H*‐(CH_3_)_2_), 1.04 (d,^3^ *J*_H,H_ = 6.7 Hz, 6H, CH‐(C*H*_*3*_)_*2*_); ^13^C NMR (75 MHz, CDCl_3_): *δ* 155.7, 148.2, 136.5, 133.1 (2C), 119.2, 117.7 (2C), 83.1, 77.7, 77.4, 27.9, 18.8 (2C); HRMS (+ESI) *m/z* [M+H]^+^ calculated for C_14_H_14_N_2_O_3_: 259.1077, found: 259.1078.

### Biological evaluation

#### Bacteria strains and growth conditions

The following bacterial reference strains were used in this study: *P. aeruginosa* PAO1 strain, *E. coli* ATCC 25922, *K. pneumoniae* ATCC 13883, and *En. cloacae* ATCC 23355. Five clinical *En. cloacae* strains collected in three hospital centers located in the south of France between March 2017 and November 2020 and characterized in a previous study (Table S1)^41^ were also tested. All bacteria were stored at −80°C in 25% (*v/v*) glycerol for cryoprotection. Bacteria were routinely grown on cation‐adjusted Mueller–Hinton (CA‐MHB; Sigma‐Aldrich, Saint‐Quentin Fallavier, France) agar plates and grown in Mueller–Hinton II broth (MHIIB; Sigma‐Aldrich) at 37°C under agitation at 180 rpm.

#### Antibiotics and adjuvant compounds

Chloramphenicol (98%), doxycycline (98%), and ampicillin (96%) reference drugs as well as Polymyxin B [USP grade] were purchased from Sigma‐Aldrich. NV716 was synthesized as previously reported^17^. Stock solutions of NV716 (10 mM) were prepared in sterile water and stored at −20°C until use.

#### Susceptibility testing on *P. aeruginosa* PAO1, *E. coli* ATCC 25922, *K. pneumoniae* ATCC 13883, and *En. cloacae* ATCC 23355

The concentrations of compound leading to 90% of bacterial growth (MIC_90_) were determined using the rapid INT colorimetric assay, as reported previously^36^, ^37^. Consistent with previous experiments with NV716 on these Gram‐negative bacteria^13^, ^15^, ^16^, ^25^, ^38^, ^39^, sub‐MIC final concentrations of this adjuvant were used (i.e., 4.1 µg/ml = 0.2× MIC_90_ for *P. aeruginosa* and *K. pneumoniae*; 1.2 µg/ml = 0.24× MIC_90_ for *E. coli* and *En. cloacae*). All experiments were performed independently in triplicate.

### Effects of NV716 on *En. cloacae* membranes—adapted from Refs.^39,40^

#### Outer membrane permeabilization assay

The permeabilization of the outer membrane of *En. cloacae* was assessed using a nitrocefin hydrolysis assay, as previously reported^40^. Potassium Phosphate Buffer (PPB, pH 7.4) was used as a negative control and Polymyxin B^25^, ^45^ was used as a positive control. Absorbance at 490 nm related to nitrocefin hydrolysis was monitored over 1 h using a Tecan Infinite® 200 Pro multimode microplate reader (Tecan Group Ltd). Experiments were performed in triplicate.

#### Inner membrane permeability assay

The inner‐membrane permeabilization by NV716 was evaluated using propidium iodide (PI, Sigma‐Aldrich), a cell‐impermeable DNA/RNA fluorescent dye, as previously described^40^. Polymyxin B^25^, ^45^ was used as a positive control and PBS buffer was used as a negative control.

#### Inner membrane depolarization assay

The inner‐membrane depolarization was evaluated using the DiSC_3_(5) (3,3'‐dipropylthiadicarbocyanine iodide, Sigma‐Aldrich) assay, as described previously^45^. Polymyxin B^25^, ^45^ was used as a positive control and HEPES‐Sucrose buffer was used as a negative control.

#### Glucose‐triggered 1,2′‐DiNA real‐time efflux assay

The efflux activity in *En. cloacae* was assessed as reported previously^24^. Maximum efflux activity (100%) was defined as the difference between the fluorescence value obtained after 620 s in the presence/absence of glucose, thereby corresponding to the efflux of 1,2'‐DiNA (TCI‐Europe SA).

#### Bacteria labeling with CyC_32‐Dansyl_

Bacterial cells (2.0 × 10^10^ cells/ml) were incubated overnight at 37°C under shaking at 180 rpm with 124 µg/ml (200 µM) of CyC_32‐Dansyl_ or DMSO (negative control) and in the presence or absence of 40.6 µg/ml (100 µM) NV716, then washed, resuspended in 250 µl PBS, and fixed with 4% paraformaldehyde. Fluorescence microscopy imaging and the analysis of Dansyl mean fluorescence intensity were performed as previously reported^35^.

### Activity‐based protein profiling

The capture of *En. cloacae* ATCC23355 potential target proteins from CyC_32yne_‐ and iBP_yne_OX‐treated culture via ABPP experiments, the mass spectrometry analysis of resulting proteomes, and the subsequent proteins identification and quantification were performed as previously described^33^.

The mass spectrometry proteomics data have been deposited to the ProteomeXchange Consortium (www.proteomexchange.org)^68^ via the PRIDE partner repository^69^ (https://www.ebi.ac.uk/pride/login) with the dataset identifiers PXD053955 for *En. cloacae* culture and PXD052024 for *P. aeruginosa* crude total lysate.

### Statistical analysis

All statistical analyses were performed using GraphPad Prism 8 (GraphPad Inc.) and are detailed in the figure legends. Differences were considered significant for calculated *p*‐values ≤ 0.05.

## AUTHOR CONTRIBUTIONS

**Emma Forest**: Investigations; formal analysis; visualization; writing—original draft; writing—review and editing. **Jordan Lehoux**: Resources; writing—review and editing. **Alexandre Guy**: Resources; writing—review and editing. **Thierry Durand**: Resources; writing—review and editing. **Stéphane Audebert**: Data curation; investigation; writing—review and editing. **Luc Camoin**: Data curation; validation; writing—review and editing. **Christopher D. Spilling**: Resources; writing—review and editing. **Céline Crauste**: Resources; writing—review and editing. **Stéphane Canaan**: Formal analysis; writing—review and editing. **Jean Michel Brunel**: Formal analysis; resources; writing—original draft; writing—review and editing. **Jean‐Michel Bolla**: Conceptualization; methodology; project administration; supervision; validation; writing—original draft; writing—review and editing. **Jean‐François Cavalier**: Conceptualization; formal analysis; methodology; project administration; supervision; validation; visualization; writing—original draft; writing—review and editing.

## ETHICS STATEMENT

This study did not involve human subjects and animals.

## CONFLICT OF INTERESTS

The authors declare no conflict of interests.

## Supporting information


**Additional file 1: Detailed protocols**; **Table S1**, Clinical characteristics of the five *En. cloacae* clinical strains; **Table S2**, Susceptibility testing of the five clinical *En. cloacae* strains to Doxycycline (DOX) and Chloramphenicol (CHL) in the presence/absence of **NV716**; **Figure S1**, growth curves of *En. cloacae* ATCC 23355 in the presence/absence of **NV716**; **Figure S2**, representative fluorescence images of *Pseudomonas aeruginosa* PAO1 strain in the presence of the fluorescent **CyC**_**32‐Dansyl**_. **Figure S3**, influence of increasing concentrations of **NV716** on membrane properties, efflux, and survival of *En. cloacae* ATCC 23355 strain; **Figure S4**, NMR spectra of the new compounds synthesized to access the **iBP**_**yne**_ **OX** probe (PDF).

**Additional file 2: Tables S3‐S5**, Target proteins identified from *En. cloacae* ATCC 23355 culture, through CC‐ABPP by LC‐ESI‐MS/MS analysis, using **iBP**_**yne**_ **OX** or **CyC**_**32yne**_ probes in the presence of **NV716**; **Table S6**, Target proteins identified from *Pseudomonas aeruginosa* PAO1 total lysate, through CC‐ABPP by LC‐ESI‐MS/MS analysis, using the **CyC**_**32yne**_ probe (XLSX).

## Data Availability

All data generated or analyzed during this study are included in this article and its supplementary information files. The mass spectrometry proteomics data are available online through the ProteomeXchange Consortium (www.proteomexchange.org) with the dataset identifiers PXD053955 for *En. cloacae* culture and PXD052024 for *Pseudomonas aeruginosa* crude total lysate.
