# Supplementary material for: The polyamino‐isoprenyl enhancer NV716 enables the antibacterial activity of two families of multi‐target inhibitors against the ESKAPEE bacterium Enterobacter cloacae
Source: mLife. 2025 Jun 25;4(3):259–74. doi: 10.1002/mlf2.70014 (PMC12207906; doi:10.1002/mlf2.70014)
Supplement: Supplementary file 1 — Additional file 1: Detailed protocols; Table S1, Clinical characteristics of the five En. cloacae clinical strains; Table S2, Susceptibility testing of the five clinical En. cloacae strains to Doxycycline (DOX) and Chloramphenicol (CHL) in the presence/absence of NV716; Figure S1, growth curves of En. cloacae ATCC 23355 in the presence/absence of NV716; Figure S2, representative fluorescence images of Pseudomonas aeruginosa PAO1 strain in the presence of the fluorescent CyC 32‐Dansyl . Figure S3, influence of increasing concentrations of NV716 on membrane properties, efflux, and survival of En. cloacae ATCC 23355 strain; Figure S4, NMR spectra of the new compounds synthesized to access the iBP yne OX probe (PDF). [file MLF2-4-259-s002.pdf]

# SUPPORTING INFORMATION

## The polyamino-isoprenyl enhancer NV716 enables the antibacterial activity of two families of multi-target inhibitors against the ESKAPEE bacterium *Enterobacter cloacae*

Emma Forest[1,2], Jordan Lehoux[3], Alexandre Guy[3], Thierry Durand[3], Stéphane Audebert[4], Luc Camoin[4], Christopher D. Spilling[5], Céline Crauste[3], Stéphane Canaan[1], Jean Michel Brunel[2], Jean-Michel Bolla[2]*, Jean-François Cavalier[1]*

[1] Aix Marseille Univ., CNRS, LISM UMR7255, IMM FR3479, Marseille, France

[2] Aix Marseille Univ, INSERM, SSA, MCT, Marseille, France

[3] IBMM, Univ Montpellier, CNRS, ENSCM, Montpellier, France

[4] Aix Marseille Univ, CNRS, INSERM, Institut Paoli-Calmettes, CRCM, Marseille Protéomique, Marseille, France

[5] Department of Chemistry and Biochemistry, University of Missouri-St. Louis, MO, USA

[*] Corresponding authors: Jean-Michel Bolla (jean-michel.BOLLA@univ-amu.fr); Jean-François Cavalier (jfcavalier@imm.cnrs.fr).

**Contents**

Detailed protocols S2

Supplementary **Tables S1-S2** S9

Supplementary **Figures S1-S3** S11

Supplementary **Figure S4**: $^{1}H$, $^{13}C$ NMR spectra of new synthesized compounds S15

References S18

# Detailed Protocols

## Chemistry

### General Methods.

All reactions were carried out with anhydrous solvents purchased from commercial sources and used without purification, unless otherwise noted. The reactions were monitored using thin layer chromatography (TLC) with pre-coated silica gel 60 plates (Merck, Kenilworth, New Jersey, USA), and visualized using a 254 nm UV lamp, stained with phosphomolybdic acid solution followed by gentle heating. The synthesized compounds were purified by column chromatography on silica gel 40–63 µm. Melting points (Mp) were determined on a Stuart capillary apparatus and are uncorrected. High resolution mass spectrometry (HRMS) was recorded using electrospray ionization (ESI) techniques on Q-TOF mass spectrometer.

**NMR Characterization.** $^{1}$H and $^{13}$C NMR spectra were recorded at 300 MHz and 75 MHz, respectively, using Bruker spectrometers. Chemical shifts are reported in parts per million (ppm, δ) relative to residual deuterated solvent peaks. The NMR spectra were assigned with the help of 2D NMR analyses. The multiplicities reported are as follows: br = broad singlet, m = multiplet, s = singlet, d = doublet, t = triplet, q = quadruplet, quint = quintuplet, h = hexuplet, or combinations thereof.

## Biological evaluation

**Bacteria strains and growth conditions.** The following bacterial reference strains were used in this study: *P. aeruginosa* PAO1 strain, *E. coli* ATCC 25922, *K. pneumoniae* ATCC 13883, and *E. cloacae* ATCC 23355. Five clinical *E. cloacae* strains collected in three hospital centers located in the south of France between March 2017 and November 2020, and characterized in a previous study (**Table S1**)[1] were also tested. All bacteria were stored at -80 °C in 25% (*v/v*) glycerol for cryoprotection. Bacteria were routinely grown on cation-adjusted Mueller-Hinton (CA-MHB; Sigma-Aldrich, Saint-Quentin Fallavier, France) agar plates and grown in Mueller-Hinton II broth (MHIIB; Sigma-Aldrich) at 37 °C under agitation at 180 rpm.

**Antibiotics and adjuvant compounds.** Chloramphenicol [98%], doxycycline [98%] and ampicillin [96%] reference drugs, as well as Polymyxin B [USP grade] were purchased from Sigma-Aldrich. **NV716** was synthesized as previously reported[2]. Stock solutions of **NV716** (10 mM) were prepared in sterile water and stored at −20 °C until use.

**Susceptibility testing on *P. aeruginosa* PAO1, *E. coli* ATCC 25922, *K. pneumoniae* ATCC 13883, and *E. cloacae* ATCC 23355.** The concentrations of compound leading to 90% of bacterial growth ($MIC_{90}$) were determined using the rapid INT colorimetric assay[3, 4]. Briefly, fresh mid-log phase culture ($OD_{600}$ = 0.6-0.8) of the different bacterial strains were diluted to a cell density of 1 × $10^{6}$ cells/mL in

MHIIB. Then, 100 µL of this inoculum was added in a 96-well flat-bottom Corning® microplates with lid (Merck, ref CLS3370) containing two-fold serial dilutions of each compound in the absence or presence of **NV716** to a final volume of 200 µL. Consistent with previous experiments with **NV716** on these Gram-negative bacteria[5-10], sub-MIC final concentrations of this adjuvant were used (*i.e.*, 4.1 µg/mL = 0.2× $MIC_{90}$ for *P. aeruginosa* and *K. pneumoniae*; 1.2 µg/mL = 0.24× $MIC_{90}$ for *E. coli* and *E. cloacae*). Growth controls (*i.e.*, bacteria only = *B*), inhibition controls with standard drugs, and sterility controls (*i.e.*, medium only) were included. Plates were incubated at 37 °C for 16-18 h, then 50 µL of an *p*-iodonitrophenyltetrazolium violet (INT) (Sigma-Aldrich) solution (0.2 mg/mL was added to each well. The microplates were re-incubated in the dark at 37 °C for about 30 min until the appearance of a color change in the control *B*-wells. Indeed, in the presence of active dehydrogenases, the colorless INT solution is reduced to an insoluble purple formazan dye. This color change, which indicates the presence of metabolically active bacteria and is a marker of cell viability, is rapid and the formazan formed is stable, so this color will not fade. The absorbance of formazans from INT was further measured at 470 nm with a Tecan Infinite® 200 PRO multimode microplate reader (Tecan Group Ltd, France). Relative absorbance units were defined as: RAU% = (test well $A_{470\ nm}$/mean $A_{470\ nm}$ of control *B* wells) × 100. MIC values were determined by fitting the RAU% sigmoidal dose−response curves in Kaleidagraph 4.2 software (Synergy Software). The drug concentration that resulted in a 90% reduction in optical density compared to control growth results was defined as the $MIC_{90}$. All experiments were performed independently in triplicate.

### Effects of NV716 on *E. cloacae* membranes - adapted from [9, 11]

***Outer membrane permeabilization assay.*** The permeabilization of the outer membrane of *E. cloacae* was assessed using a nitrocefin hydrolysis assay[11]. Nitrocefin is a chromogenic cephalosporin which is converted by β-lactamases in a red-colored derivative in the periplasmic space of permeabilized bacteria[12]. Therefore, the permeabilization of the outer membrane can be monitored by following the absorbance at 490 nm (red). Fresh bacterial culture grown up to $OD_{600\ nm}$ = 0.3, was incubated with 0.25 µg/mL imipenem for 2 h at 37 °C to induce β-lactamase expression[13]. The culture was then pelleted at 3,000 *g* for 10 min, washed two times with Potassium Phosphate Buffer (PPB, pH 7.4), and resuspended at a cell density of 4.7 × $10^8$ cells/mL in PPB (*i.e.*, $OD_{600}$ ~ 0.4). One hundred µL of the latter bacterial solution were mixed with 50 µL of various concentrations of **NV716** in a 96-well translucent flat-bottom Corning® microplate with lid (Merck, ref. CLS3370), then 50 µL of nitrocefin solution (ThermoFischer Scientific, ref. SR0112C, rehydrated following kit instructions and diluted with 3 mL of PPB-5% DMSO - final concentration in the well 50 µg/mL) were added. PPB was used as negative control, and Polymyxin B[10, 14] as positive control. Absorbance at 490 nm related to nitrocefin hydrolysis was monitored over 60 min with an interval of 1 min using a Tecan Infinite® 200 Pro multimode microplate reader (Tecan Group Ltd, France). Experiments were performed in triplicate.

***Inner membrane permeability assay.*** The inner membrane permeabilization by **NV716** was evaluated using propidium iodide (PI, Sigma Aldrich), a cell-impermeable DNA/RNA fluorescent dye, as previously described[11]. Fresh bacterial culture grown up to $OD_{600\ nm}$ of 0.4 was centrifuged at 3,000 *g* for 10 min, and resuspended at a cell density of $1.2 \times 10^8$ cells/mL in PBS (i.e., $OD_{600\ nm}$ ~ 0.1); after which PI (10 µM final concentration) was added. Ninety µL of the resulting PI-containing bacterial suspension were added in a 96-well black microplate (Greiner Bio-One, ref. 675077) together with 10 µL of various concentrations of **NV716**, and left in the dark for 1 h. The fluorescence intensity ($\lambda_{ex}$ / $\lambda_{em}$ = 530 / 610 nm) was measured using a Tecan Infinite® 200 Pro multimode microplate reader (Tecan Group Ltd, France). Polymyxin B[10, 14] was used as positive control and PBS Buffer as negative control.

***Inner membrane depolarization assay.*** The inner membrane depolarization was evaluated by using $DiSC_3(5)$ (3,3'-dipropylthiadicarbocyanine iodide – Sigma Aldrich) assay as described previously[14]. Fresh bacterial culture grown up to $OD_{600\ nm}$ of 0.4 was centrifuged at 3,000 *g* for 10 min, washed (3× PBS) and resuspended in HEPES-Sucrose Buffer (HEPES 5 mM, Sucrose 250 mM, $MgSO_4$ 25 mM) at a final cell density of $1.2 \times 10^8$ cells/mL (*i.e.*, $OD_{600\ nm}$ ~ 0.1) (final volume 10 mL); after which $DiSC_3(5)$ (10 µM final concentration) was added. Ninety µL of the resulting bacterial-$DiSC_3(5)$ solution were added in a 96-well black microplate (Greiner Bio-One, ref. 675077), and left in the dark at 37 °C for 1 h. The membrane potential-sensitive cyanine dye $DiSC_3(5)$ accumulates on polarized membranes resulting in self-quenching of fluorescence[15]. Upon membrane depolarization, the dye is released and fluorescence de-quenched. Released $DiSC_3(5)$ was quantified 300 s after the addition of various concentrations of **NV716** (10 µL) by measuring the fluorescence intensity ($\lambda_{ex}$ / $\lambda_{em}$ = 654 / 675 nm) over 30 min with an interval of 1 min, using a Tecan Infinite® 200 Pro multimode microplate reader (Tecan Group Ltd, France). Polymyxin B[10, 14] was used as positive control and HEPES-Sucrose Buffer as negative control.

***Glucose-triggered 1,2′-diNA real-time efflux assay.*** The efflux activity in *E. cloacae* was assessed as reported previously[16]. Ten milliliters of overnight culture in MHIIB were centrifuged at 3,000 *g* for 7 min, washed once with PBS, and then incubated in PBS for 3 h at 37 °C under gentle agitation with 10 µM 1,2′-dinaphthylamine (1,2-DiNA - TCI-Europe SA, Zwijndrecht, Belgium) and 5 µM carbonyl cyanide *m*-chlorophenylhydrazone (CCCP – Sigma Aldrich), to de-energize the efflux pumps. The cells were centrifuged at 3,000 *g* for 10 min, resuspended in PBS and adjusted to a cell density of $5.8 \times 10^8$ cells/mL ($OD_{600\ nm}$ ~ 0.5). Ninety µL of bacterial suspension were added in a 96-well black microplate (Greiner Bio-One, ref 675077) before addition of 10 µL of various concentrations of **NV716**. The fluorescence intensity ($\lambda_{ex}$ / $\lambda_{em}$ = 370 / 420 nm) was monitored each 30 s during 900 s using a Tecan Infinite® 200 Pro multimode microplate reader (Tecan Group Ltd, France). Active efflux was induced by the addition of 10 µL glucose (100 mM final concentration) at 180 s. Maximum efflux activity (100%) was defined as the difference between the fluorescence value obtained after 620 s in the presence / absence of glucose.

**Bacteria labelling with CyC$_{32\text{-Dansyl}}$ – adapted from** [17]. Mid-log phase bacterial suspension ($OD_{600\ nm}$ = 0.4-0.6) in MHIIB was pelleted for 10 min at 3,000 *g* and resuspended in 250 µL of MHII at a theoretical $OD_{600\ nm}$ of 20 corresponding to 2.0 × $10^{10}$ cells/mL. The cells were then incubated overnight at 37 °C under shaking at 180 rpm with 124 µg/mL (= 200 µM) of **CyC$_{32\text{-Dansyl}}$** or DMSO (negative control) and in the presence or absence of 40.6 µg/mL (= 100 µM) **NV716**. After incubation, bacteria were washed three times with PBS and resuspended in 250 µL PBS. For microscopy observation, bacteria were fixed with 4% paraformaldehyde in PBS for 30 min at room temperature, washed 3 times with PBS and resuspend in 250 µL of PBS.

**Fluorescence microscopy – adapted from** [17]. Fixed samples (10 µL bacterial suspension, *i.e.*, 2.0 × $10^8$ cells), spotted on an agar pad between a microscope slide and coverslip were analyzed by snapshot fluorescence imaging at room temperature using an Olympus IX81 confocal microscope equipped with a UPlanSApo 100× 1.40 NA objective and operated with the FV1000 software. Exposure time was 10 µs/pixel for both laser transmission and fluorescence images with conserved settings (X, Y, Z, HV, Gain and offset). The Dansyl was detected by excitation with a 405 nm laser with 39-50% intensity, and emission was collected using a semitransparent mirror collecting all wavelength under 560 nm. Three biological replicates were made for each condition, and for each sample different images were taken per frame in order to get 150 bacteria in total. Images were next converted to an 8-bit intensity color range. In all cases, the corresponding control images were taken/checked prior to any fluorescence imaging and quantification.

**Analysis of Dansyl mean fluorescence intensity**. A background noise was first calculated by taking the mean fluorescence intensity of 10 different areas free of any bacteria and was subtracted to the frame. Segmentation and analysis of the images recorded (63.49 × 63.49 µm; 512 × 512 pixels) were performed using the open-source program ImageJ/Fiji 1.53f51. Cells segmentation was done by manually detouring the bacteria on the laser transmission frame and transforming it into a mask. This mask was then used to determine the Dansyl mean fluorescence intensity of pixels associated with the bacterial region corresponding to the **CyC$_{32\text{-Dansyl}}$** bacterial uptake.

**Activity-based protein profiling – adapted from** [18]

***Capture of E. cloacae ATCC23355 potential target proteins from CyC$_{32yne}$ and iBP$_{yne}$OX treated culture via ABPP experiments.*** Mid-log phase *E. cloacae* ATCC 23355 bacterial suspension ($OD_{600\ nm}$ = 0.4-0.6) in MHIIB was pelleted for 10 min at 3,000 *g* and resuspended in 1 mL of MHII at a theoretical $OD_{600\ nm}$ of 20 (*i.e.*, 2.0 × $10^{10}$ cells/mL). The bacterial cells were then incubated overnight at 37 °C under shaking at 180 rpm with **CyC$_{32yne}$** (74 µg/mL = 200 µM final concentration), **iBP$_{yne}$OX** (129 µg/mL = 500 µM final concentration) or DMSO (control) in the presence of **NV716** (40.6 µg/mL = 100 µM final concentration). Bacteria were washed 3 times with PBS containing 0.05% Tween 80, and

resuspended in PBS supplemented with EDTA-free protease inhibitors (cOmplete Mini, EDTA-free; Roche, Mannheim, Germany) at a 1:1 (*w/v*) ratio. The bacterial cells were mixed with 200 µL of 0.1 mm diameter glass beads (BioSpec) in 2 mL Eppendorf tubes and disrupted during 2 × 4 min of violent shaking, with ice cooling between each run, using Mini-Beadbeater-96 (BioSpec, Bartlesville, OK, USA). The lysate was cooled down in ice for 5 min and then centrifuged at 4 °C and at 200 *g* for 10 min to remove the cell debris and unbroken cells. The concentration of total proteins in the supernatants was determined *via* the Bradford method, and adjusted to a concentration of 1 mg/mL.

Both **CyC$_{32yne}$-** and **iBP$_{yne}$OX-**treated *E. cloacae* and DMSO-control samples (300 µL – 0.3 mg total proteins) were subjected to click-chemistry reaction by the successive addition of Desthiobiotin-PEG$_3$-N$_3$ (Jena Bioscience, Jena, Germany, CLK-AZ104P4–100), TBTA ligand and freshly prepared TCEP solution (Sigma-Aldrich) to the cells (see table below for specific added volumes).

| **Reagents** | **Stock solution** | **Volume of reagent (µL) depending on the probe concentration 74 µg/mL / 129 µg/mL** |
|---|---|---|
| Desthiobiotin-Peg$_3$-N$_3$ | 40 mM DMSO | 3.2 / 8.0 |
| TCEP | 50 mM $H_2O$ | 6.3 / 16.0 |
| TBTA | 1.667 mM *t*BuOH/DMSO (80:20, *v/v*) | 19.0 / 48.1 |
| $CuSO_4$ | 50 mM $H_2O$ | 6.3 / 16.0 |

After gently vortexing of the samples, the cycloaddition reaction was initiated by addition of $CuSO_4$ solution (Sigma-Aldrich). The lysates were mixed by vortexing again and incubated for 2 h under gentle rotative agitation (15 rpm) at room temperature in the dark. The proteins were further precipitated overnight at −20 °C by adding 4 mL ice-cold acetone, and pelletized by centrifugation (16,200 *g*, 4 °C, 15 min). The supernatant was discarded, and the proteins were washed with 2 × 500 µL ice-cold MeOH (resuspension by sonication, 1 × 10 s, 10% max. intensity). After centrifugation (16,200 *g*, 4 °C, 15 min), the pellets were resuspended in 0.2% (*w/v*) SDS in PBS (250 µL) at room temperature by mild sonication (4 × 10 s, 10% max. intensity). Affinity enrichment was performed with 25 µL Pierce High Capacity Streptavidin Agarose Resin (ThermoFischer Scientific, ref. 20359; prewashed 3 × 250 µL 0.4% (*w/v*) SDS solution and 3 × 250 µL PBS, centrifugation was performed at 500 *g* for 2 min). Each *E. cloacae* treated-lysate was enriched for labelled proteins by transfer to the previously washed beads (around 250 µg) and incubated under gentle rotative agitation (15 rpm) at room temperature for 3 h. Beads were stringently washed following pull-down (500 µL each time): 2× 0.4% (*w/v*) SDS in PBS, and 1× 10 M urea in PBS. All centrifugation steps were conducted at 500 *g* for 2 min at room temperature. The beads containing bound, biotinylated proteins were resuspended in 50 µL PBS buffer pH 7.4 containing 50 mM free D-biotin. The resulting solution was mixed with 5X Laemmli reducing

sample buffer, and heated at 95 °C for 5 min. Next, the samples were snap frozen in liquid nitrogen and stored at −80 °C before mass spectrometry experiments. To exclude any unspecific binding, the DMSO-treated lysate sample was also incubated with the streptavidin-agarose beads, and processed as described above.

***Mass spectrometry analysis.*** The protein isolates were loaded on NuPAGE™ 4-12% Bis–tris acrylamide gels according to the manufacturer's instructions (Invitrogen, Life Technologies). Running of samples was stopped as soon as proteins stacked as a single band. Protein containing bands were stained with Thermo Scientific Imperial Blue, cut from the gel, and following reduction and iodoacetamide alkylation, digested with high sequencing grade trypsin (Promega, Madison, WI, USA) [18-20]. Extracted peptides were concentrated before mass spectrometry analysis under speed-vacuum. Samples were reconstituted with 0.1% trifluoroacetic acid in 2% acetonitrile and analyzed by liquid chromatography (LC)-tandem MS (MS/MS) using a Q Exactive Plus Hybrid Quadrupole-Orbitrap online with a nanoLC Ultimate 3000 chromatography system (Thermo Fisher Scientific™, San Jose, CA). For each biological sample, 4 µL corresponding to 26.6% of digested sample were injected in duplicate on the system. After pre-concentration and washing of the sample on a Acclaim PepMap 100 column (C18, 2 cm × 100 µm i.d. 100 Å pore size, 5 µm particle size), peptides were separated on a LC EASY-Spray column (C18, 50 cm × 75 µm i.d., 100 Å, 2 µm, 100 Å particle size) at a flow rate of 300 nL/min with a two-step linear gradient (2-22% acetonitrile/$H_2$0; 0.1% formic acid for 100 min and 22-32% acetonitrile/$H_2$0; 0.1% formic acid for 20 min). For peptides ionization in the EASY-Spray source, spray voltage was set at 1.9 kV and the capillary temperature at 250 °C. All samples were measured in a data dependent acquisition mode. Each run was preceded by a blank MS run in order to monitor system background. The peptide masses were measured in a survey full scan (scan range 375-1500 *m/z*, with 70 K FWHM resolution at *m/z*=400, target AGC value of $3.00\times10^6$ and maximum injection time of 100 ms). Following the high-resolution full scan in the Orbitrap, the 10 most intense data-dependent precursor ions were successively fragmented in HCD cell and measured in Orbitrap (normalized collision energy of 25%, activation time of 10 ms, target AGC value of $1.00\times10^5$, intensity threshold $1.00\times10^4$ maximum injection time 100 ms, isolation window 2 *m/z*, 17.5 K FWHM resolution, scan range 200 to 2,000 *m/z*). Dynamic exclusion was implemented with a repeat count of 1 and exclusion duration of 20 s.

***Protein identification and quantification.*** Relative intensity-based label-free quantification (LFQ) was processed using the MaxLFQ algorithm[21] from the freely available MaxQuant computational proteomics platform, version 1.6.3.4[22]. Analysis was done on three biological duplicates, each injected two times on mass spectrometers. The acquired raw LC Orbitrap MS data were first processed using the integrated Andromeda search engine[23]. Spectra were searched either against the *E. cloacae* (UP000002363) database extracted from UniProt (5708 entries), or the *P. aeruginosa* Swiss-Prot Reviewed database extracted from UniProt (3665 entries)[24]. The false discovery rate (FDR) at the

peptide and protein levels were set to 1% and determined by searching a reverse database. For protein grouping, all proteins that could not be distinguished based on their identified peptides were assembled into a single entry according to the MaxQuant rules. The statistical analysis was done with Perseus program (version 1.6.15) from the MaxQuant environment (www.maxquant.org). Quantifiable proteins were defined as those detected in above 70% of samples in one condition or more. Protein LFQ normalized intensities were base 2 logarithmized to obtain a normal distribution. Missing values were replaced using data imputation by randomly selecting from a normal distribution centered on the lower edge of the intensity values that simulates signals of low abundant proteins using default parameters (a downshift of 1.8 standard deviation and a width of 0.3 of the original distribution). To determine whether a given detected protein was specifically differential in **CyC$_{32yne}$** or **iBP$_{yne}$OX** pulldown, a two-sample *t*-test was done using permutation-based FDR-controlled at 5 and employing 250 permutations. The *p*-value was adjusted using a scaling factor s0 with a value of 1[25].

The mass spectrometry proteomics data have been deposited to the ProteomeXchange Consortium (www.proteomexchange.org)[26] *via* the PRIDE partner repository[27] (https://www.ebi.ac.uk/pride/login) with the dataset identifiers PXD053955 for *E. cloacae* culture; and PXD052024 for *P. aeruginosa* crude total lysate.

**Table S1.** Clinical characteristics of the five *E. cloacae* clinical strains collected between Oct-2017 and May-2020 in three hospital centers in the south of France [1].[a]

| Name | Sex | Age | Collection Date | Sample | Service | Resistance phenotype | Efflux phenotype |
|---|---|---|---|---|---|---|---|
| ***Ecl53*** | M | 62 | Oct-2017 | Wound | Medicine – Aubagne HC | MDR, FQs resistant, EBSL, Carba$^R$ | Overexpression of the AcrAB efflux pump |
| ***Ecl71*** | M | 80 | Mar-2020 | Urine | La Ciotat hospital laboratory | WT | Basal |
| ***Ecl80*** | M | 77 | Feb-2020 | Blood culture | La Ciotat hospital laboratory | MDR, HLCR | Overexpression of OqxAB efflux pump |
| ***Ecl93*** | M | 89 | May-2020 | Surgical wound | Cardiology - Aubagne HC | MDR, HLCR, Carba$^R$ | Basal |
| ***Ecl136*** | M | 90 | Mar-2020 | Intraoperative sampling | Orthopedic surgery - Nîmes HC | Cephalosporinase, FQs resistant | Overexpression of the AcrAB efflux pump |

[a] Legends: M = male; *Ecl* = *E. cloacae*; MDR = multi drug resistant; EBSL = extended-spectrum beta-lactamase; FQs = fluoroquinolones; HLCR = high level cephalosporinase resistance; Carba$^R$ = resistance by impermeability to carbapenems; WT = wild type; HC = hospital center, ICU: Intensive care Unit.

**Table S2.** Susceptibility testing of the five *E. cloacae* clinical strains to Doxycycline (DOX) and Chloramphenicol (CHL) in the presence / absence of **NV716** [a]

| ***E. cloacae* strains** | **$MIC_{90}$ (µg/mL)** | | | | | |
|---|---|---|---|---|---|---|
| | **DOX** | | **Gain in MIC** | **CHL** | | **Gain in MIC** |
| | **-NV716** | **+NV716** (1.2 µg/mL) | | **-NV716** | **+NV716** (1.2 µg/mL) | |
| **ATCC 23355** | 7.5 ± 0.30 | 0.16 ± 0.01 | ×46.9 | 11 ± 0.6 | 1.7 ± 0.6 | ×6.5 |
| ***Ecl53*** | 12.8 ± 0.26 | 3.5 ± 0.12 | ×3.7 | 19.3 ± 1.5 | 1.5 ± 0.13 | ×12.9 |
| ***Ecl71*** | 11.9 ± 4.6 | 1.1 ± 0.55 | ×10.8 | 15.0 ± 9.3 | 0.82 ± 0.30 | ×18.3 |
| ***Ecl80*** | 1.7 ± 0.14 | 0.58 ± 0.08 | ×2.9 | 2.6 ± 0.22 | 1.1 ± 0.14 | ×2.4 |
| ***Ecl93*** | 1.2 ± 0.11 | 0.35 ± 0.03 | ×3.4 | 1.9 ± 0.53 | 0.51 ± 0.21 | ×3.7 |
| ***Ecl136*** | 11.0 ± 1.6 | 2.3 ± 0.32 | ×4.8 | > 64 | > 64 | - |

[a] Experiments were performed as described in **Material and Method** section. $MIC_{90}$: compound minimal concentration leading to 90% of growth inhibition, as determined by the INT assay following absorbance reading at 470 nm. Values are mean of at least three independent assays.

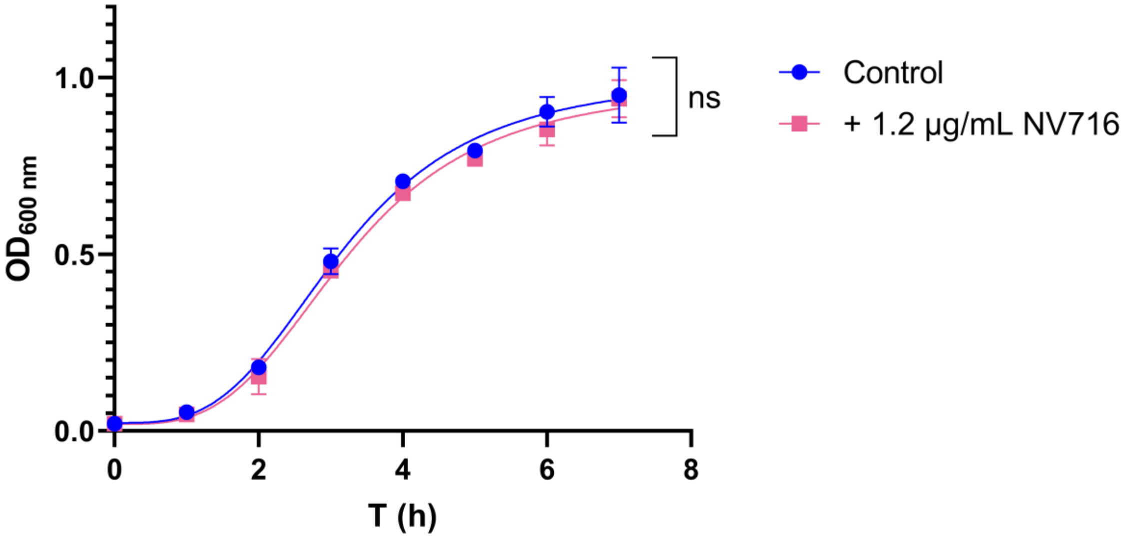


**Figure S1: Growth curves of *E. cloacae* ATCC 23355 in the presence or absence of 1.2 µg/mL NV716.** Media were inoculated at initial $OD_{600nm}$ = 0.02, and growth was followed over an 8 h period. Each data point is the mean ± SD of three biologically independent experiments. Statistical analysis was done using a non-parametric Mann–Whitney test with Prism 8.0 (GraphPad, Inc): *ns*, not significant (*p*-value >0.05).

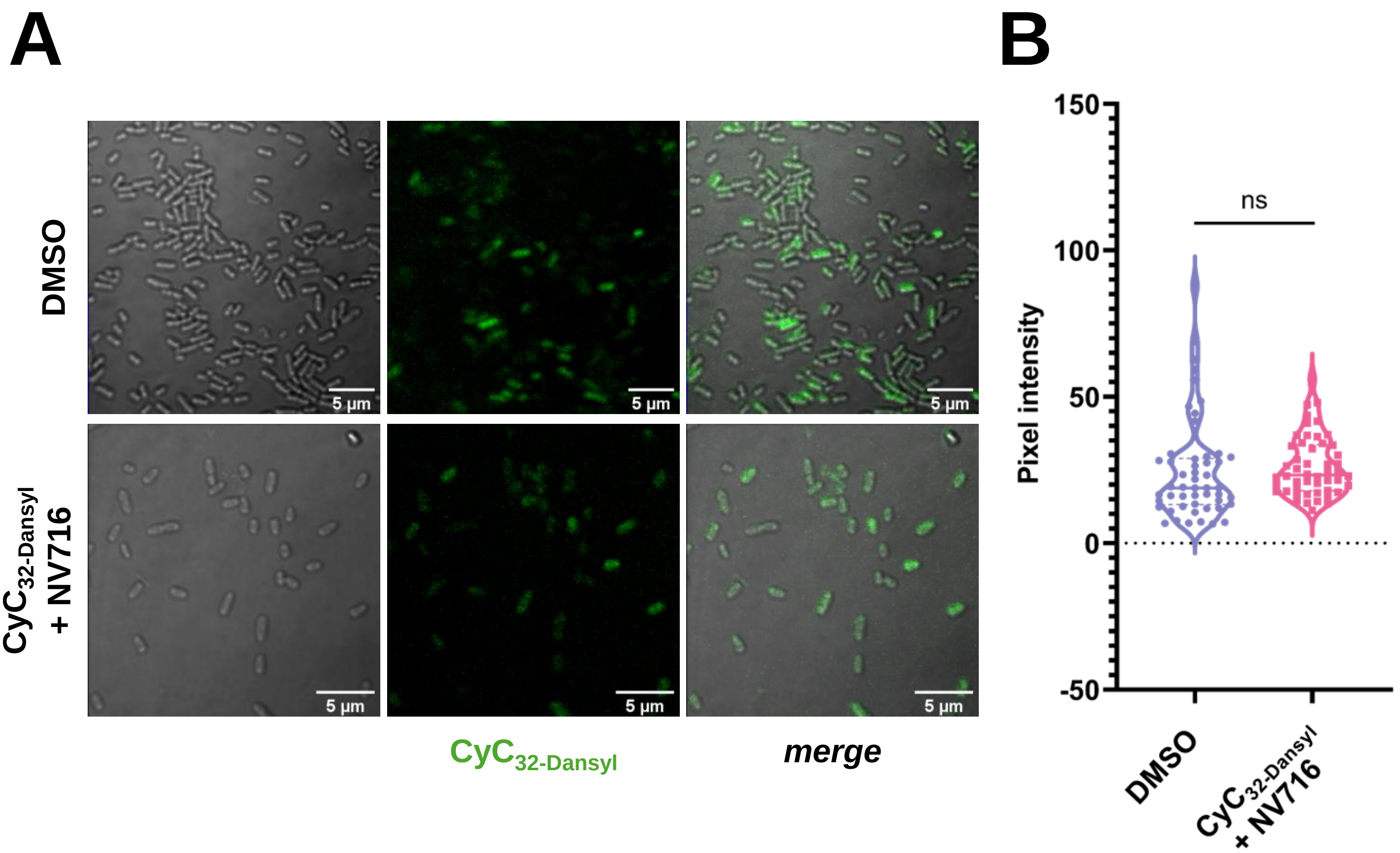


**Figure S2. CyC$_{32\text{-Dansyl}}$ is unable to accumulate in *P. aeruginosa* PAO1 despite the presence of NV716. (A)** Representative fluorescence images of *P. aeruginosa* PAO1 strain in the presence of fluorescent **CyC$_{32\text{-Dansyl}}$**. The bacteria were exposed to either 124 µg/mL **CyC$_{32\text{-Dansyl}}$** + 40.6 µg/mL **NV716**, or DMSO (vehicle, negative control), then washed and fixed with 4% paraformaldehyde. Fixed bacteria were imaged using an Olympus IX81 confocal microscope. Scale bars: 5 µm. **(B)** Quantitative analysis of the Dansyl fluorescence signal per bacterium shown as a violin plot and expressed as pixel intensity arbitrary units (au). The fluorescence seen in the control condition is due to the auto-fluorescence of *P. aeruginosa* that is overlapping with the Dansyl fluorescence. Results are from three biologically independent experiments, *n*=50 for each condition. Statistical analysis was performed by using a two-tailed *t*-statistic test with Prism 8.0 (Graphpad, Inc): *ns*, not significant (*p*-value >0.05).

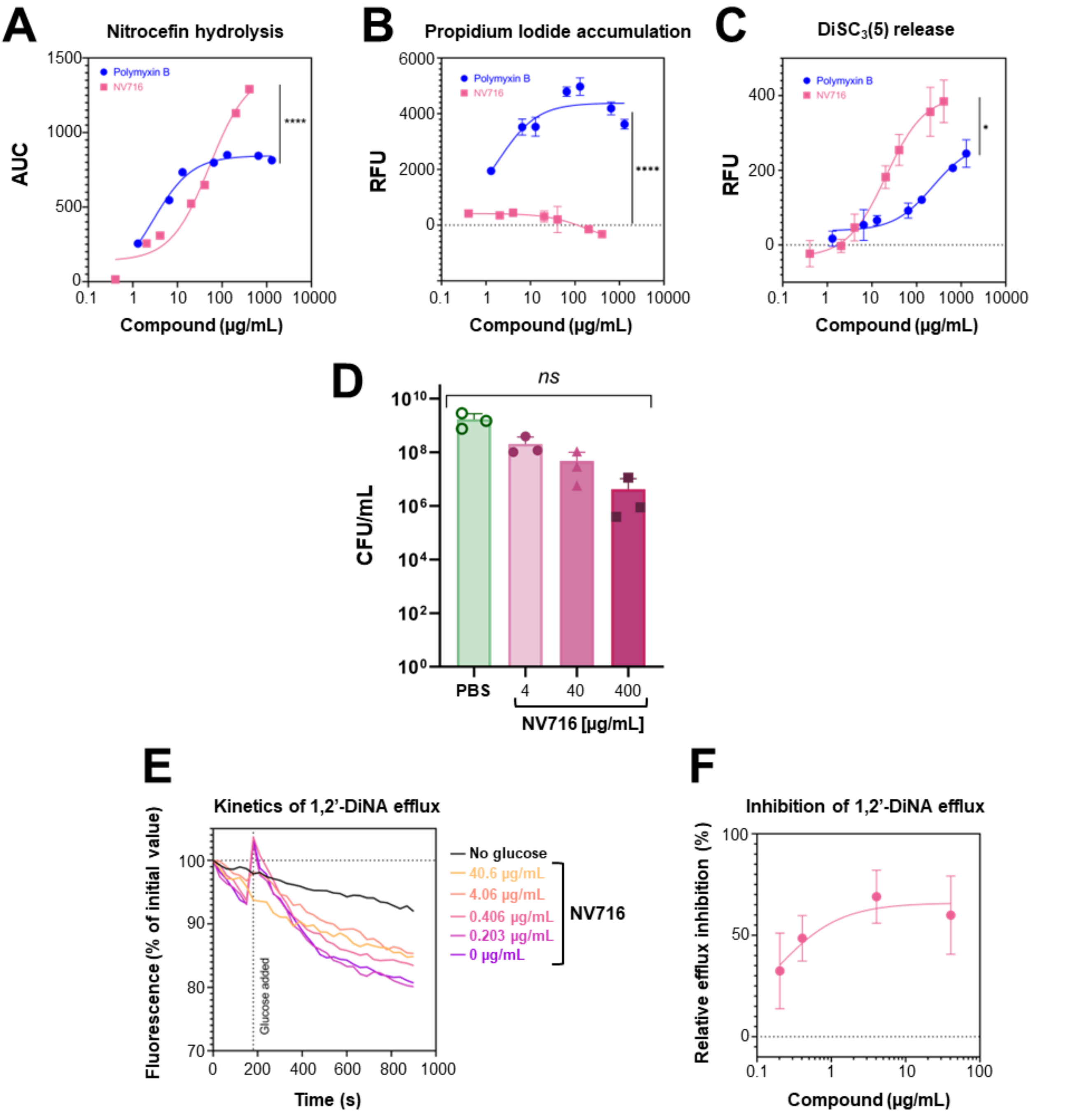


**Figure S3**. **Influence of increasing concentrations of NV716 on membrane properties, efflux and survival of *E. cloacae* ATCC 23355 strain. (A-C)** Influence of increasing concentrations of NV716 on membrane properties of *E. cloacae* ATCC 23355**. (A)** Outer membrane (OM) permeability, evaluated by measuring the absorbance at 490 nm of the hydrolysis product of nitrocefin after 1 h of incubation with **NV716** – adapted from [11]. **(B)** Outer (OM) and inner membrane (IM) integrity, evaluated by measuring the fluorescence of propidium iodide (PI) after 1 h of incubation with **NV716** – adapted from [11]. **(C)** Inner membrane (IM) polarization, assessed by monitoring the release of fluorescent $DiSC_3(5)$ in presence of **NV716** – adapted from [14]. In each test, Polymyxin B was used as positive control; and the buffer as negative control. Results are expressed as mean values ± SD of three biologically independent experiments. Statistical significance was assessed with multiple *t*-test analysis using Prism 8.0 (GraphPad, Inc): * *p*-value <0.05; ** *p*-value <0.01; **** *p*-value <0.0001. **(D)** Survival of *E. cloacae* ATCC 23355 in the presence of various concentrations of NV716**.** *E. cloacae* ATCC 23355 cultures at initial $OD_{600nm}$ ~ 0.1 (*i.e.*, 1.17 × $10^8$ cells/mL) were incubated in the presence of 4, 40 or 400 µg/mL **NV716** for 2 h and then processed for CFU count. Results are expressed as mean values ± SD of three biologically independent experiments. Statistical significance was assessed with one-way ANOVA followed by

Tukey's post hoc test using Prism 8.0 (GraphPad, Inc): *ns*, not significant (*p*-value >0.05). **(E-F)** Effect of increasing concentrations of NV716 on *E. cloacae* ATCC 23355 efflux – adapted from [16]**. (E)** Typical kinetics of DiNA efflux from *E. cloacae* in the presence of increasing concentrations of **NV716**. Active efflux was triggered by the addition of 100 mM Glucose (black vertical dotted line). **(F)** Relative DiNA efflux inhibition by **NV716**. The graph shows the percentage of efflux inhibition after 900 s of incubation (= 720 s after the addition of glucose; black vertical dotted line on the left graph). Data are expressed as mean ± SD of three independent experiments performed in triplicate. Statistical analysis assessed with multiple *t*-test analysis using Prism 8.0 (GraphPad, Inc) showed no significand difference between each data point.

**Figure S4.** $^{1}$H and $^{13}$C NMR spectra

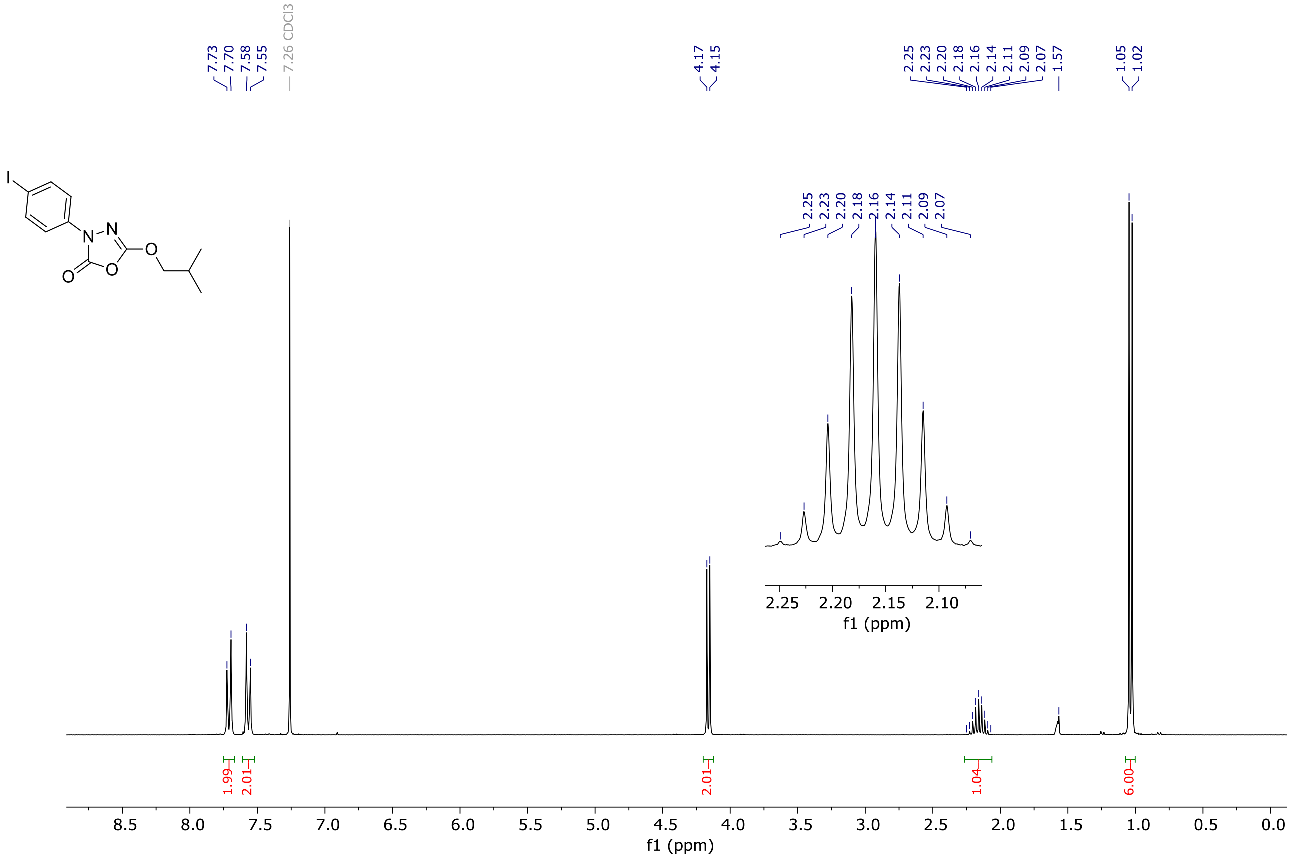


$^{1}$H NMR Spectrum (300 MHz, $CDCl_3$)

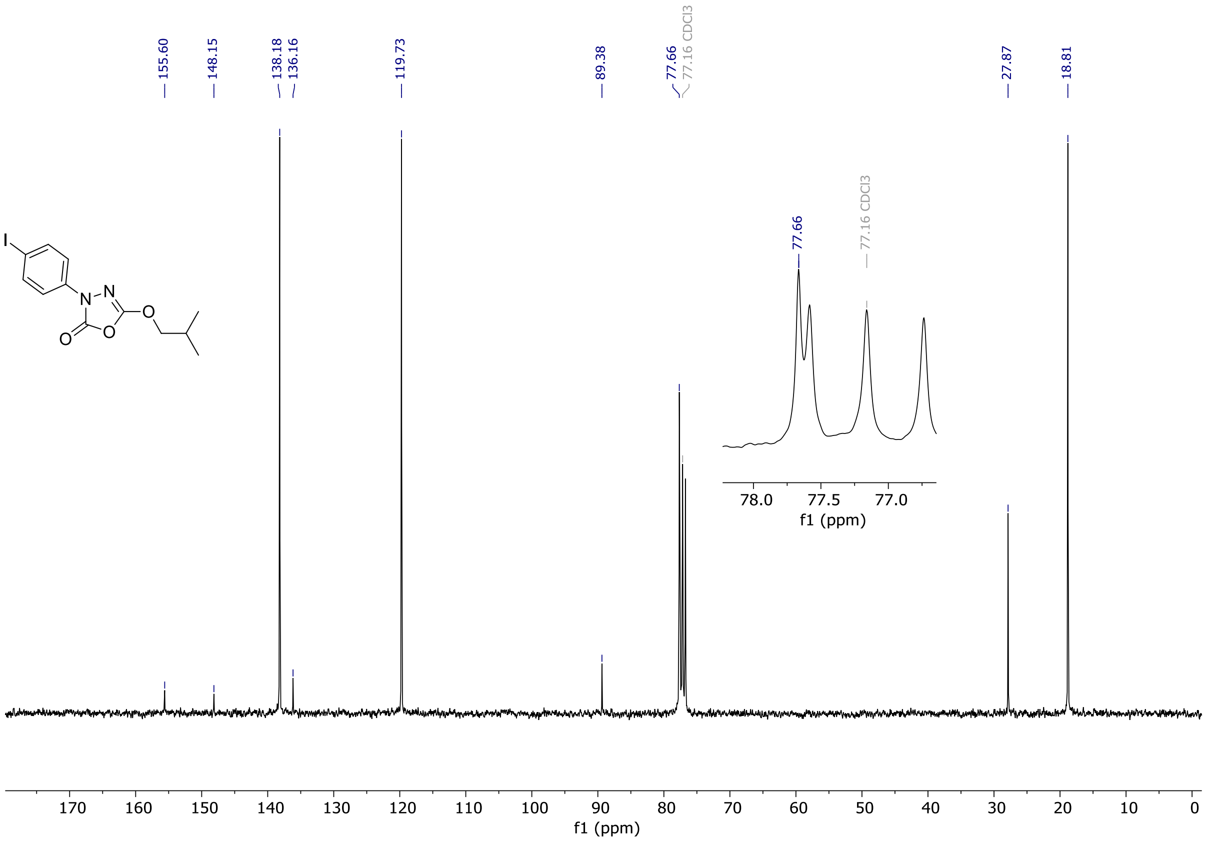


$^{13}$C NMR Spectrum (75 MHz, $CDCl_3$)

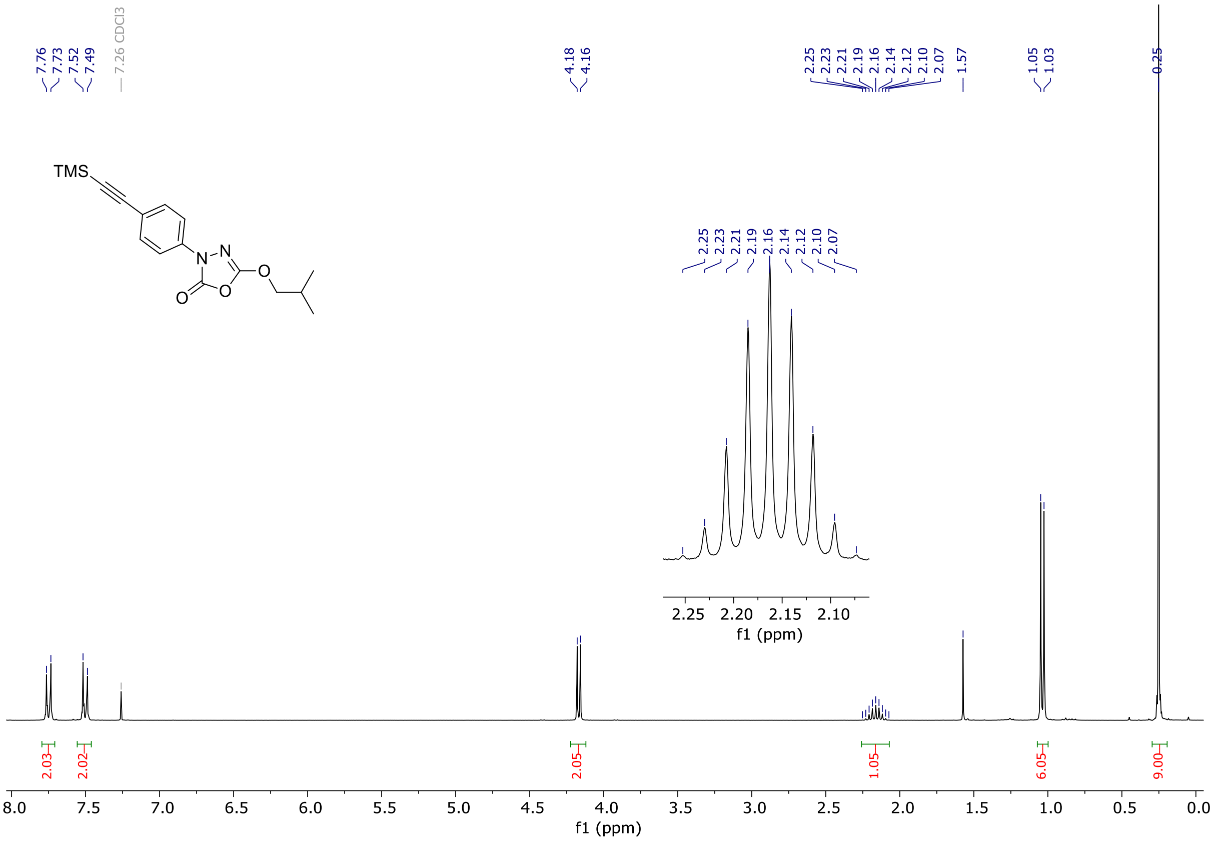


$^{1}$H NMR Spectrum (300 MHz, $CDCl_3$)

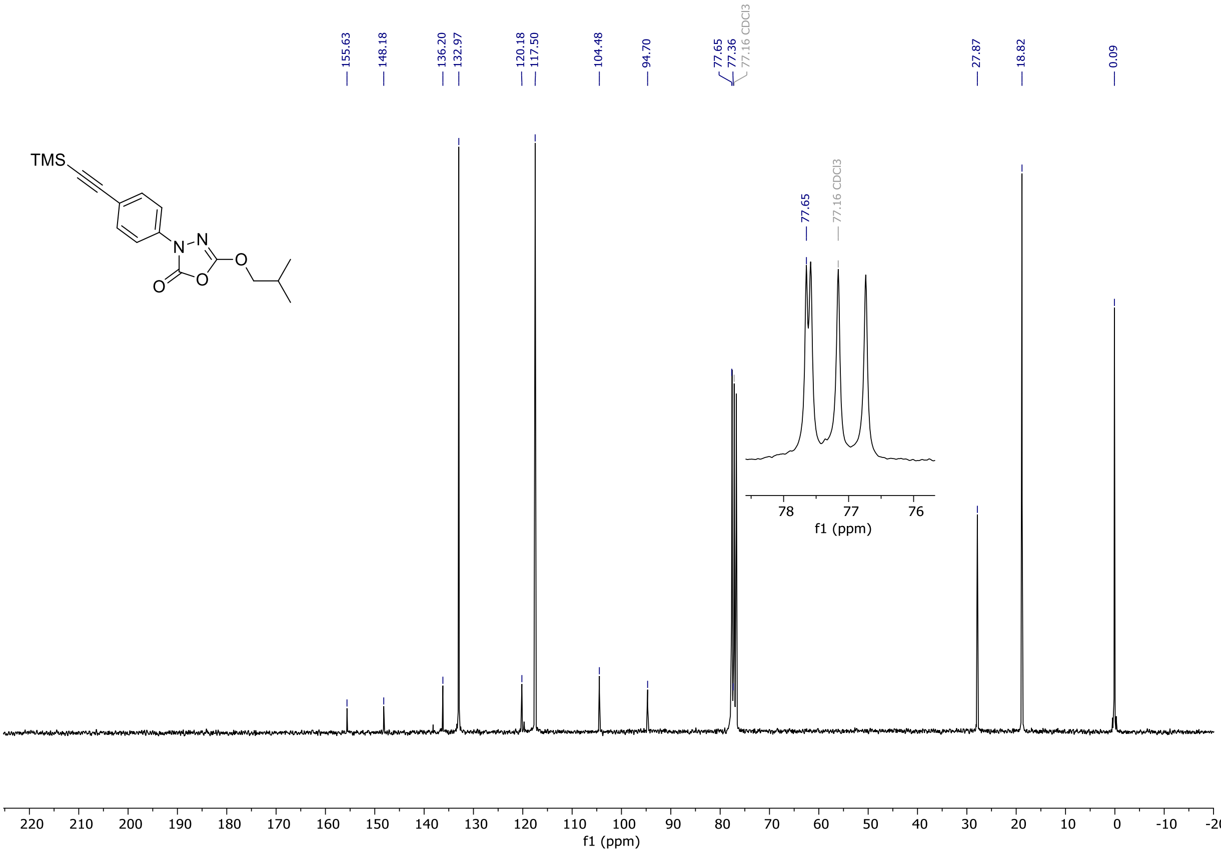


$^{13}$C NMR Spectrum (75 MHz, $CDCl_3$)

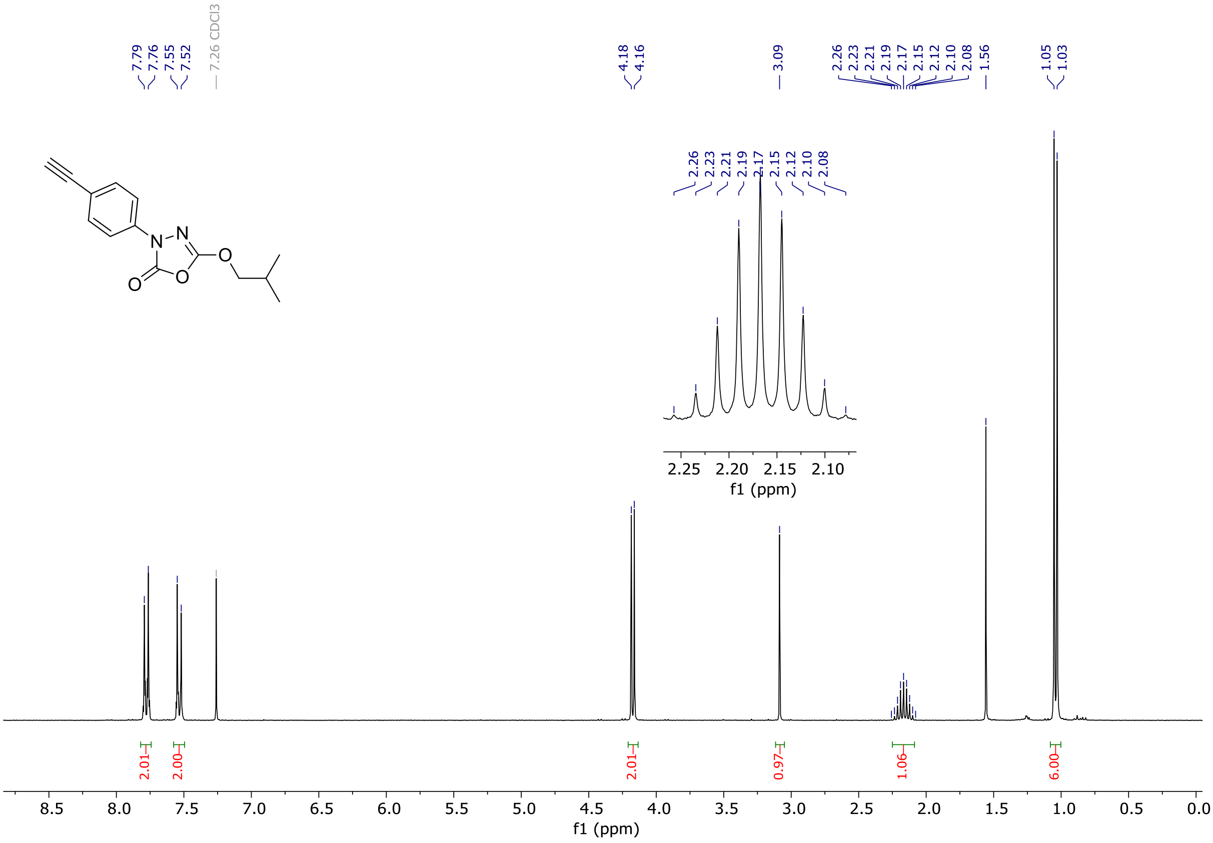


$^{1}$H NMR Spectrum (300 MHz, $CDCl_3$)

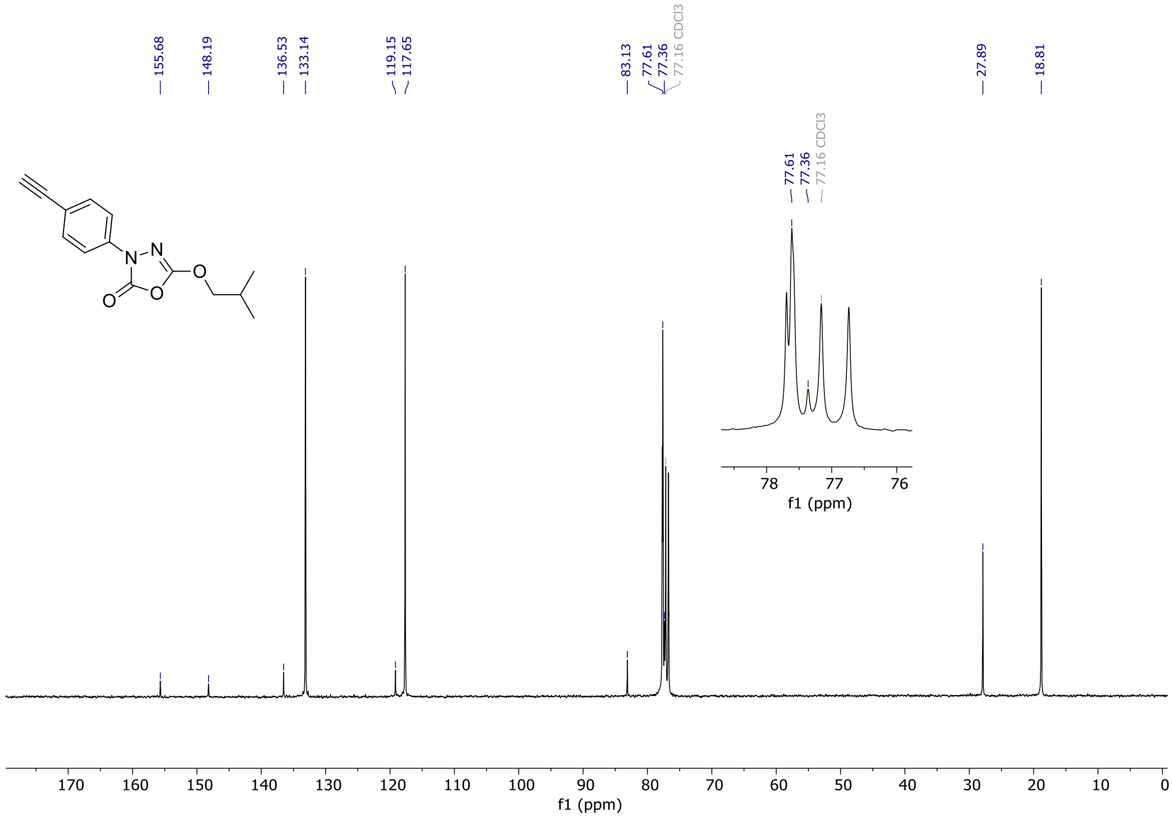


$^{13}$C NMR Spectrum (75 MHz, $CDCl_3$)

**References**


1. Ferrand A, Vergalli J, Bosi C, Pantel A, Pages JM, Davin-Regli A. Contribution of efflux and mutations in fluoroquinolone susceptibility in MDR enterobacterial isolates: a quantitative and molecular study. J Antimicrob Chemother. 2023;78(6):1532-42.

2. Lieutaud A, Pieri C, Bolla JM, Brunel JM. New Polyaminoisoprenyl Antibiotics Enhancers against Two Multidrug-Resistant Gram-Negative Bacteria from Enterobacter and Salmonella Species. J Med Chem. 2020;63(18):10496-508.

3. Weseler A, Geiss HK, Saller R, Reichling J. A novel colorimetric broth microdilution method to determine the minimum inhibitory concentration (MIC) of antibiotics and essential oils against Helicobacter pylori. Pharmazie. 2005;60(7):498-502.

4. Tunney MM, Ramage G, Field TR, Moriarty TF, Storey DG. Rapid colorimetric assay for antimicrobial susceptibility testing of Pseudomonas aeruginosa. Antimicrob Agents Chemother. 2004;48(5):1879-81.

5. Borselli D, Brunel JM, Gorge O, Bolla JM. Polyamino-Isoprenyl Derivatives as Antibiotic Adjuvants and Motility Inhibitors for Bordetella bronchiseptica Porcine Pulmonary Infection Treatment. Front Microbiol. 2019;10:1771.

6. Dhanda G, Acharya Y, Haldar J. Antibiotic Adjuvants: A Versatile Approach to Combat Antibiotic Resistance. ACS Omega. 2023;8(12):10757-83.

7. Douafer H, Andrieu V, Phanstiel Ot, Brunel JM. Antibiotic Adjuvants: Make Antibiotics Great Again! J Med Chem. 2019;62(19):8665-81.

8. Pieren M, Tigges M. Adjuvant strategies for potentiation of antibiotics to overcome antimicrobial resistance. Curr Opin Pharmacol. 2012;12(5):551-5.

9. Troudi A, Bolla JM, Klibi N, Brunel JM. An Original and Efficient Antibiotic Adjuvant Strategy to Enhance the Activity of Macrolide Antibiotics against Gram-Negative Resistant Strains. Int J Mol Sci. 2022;23(20):12457.

10. Troudi A, Fethi M, Selim El Asli M, Bolla JM, Klibi N, Brunel JM. Efficiency of a Tetracycline-Adjuvant Combination Against Multidrug Resistant Pseudomonas aeruginosa Tunisian Clinical Isolates. Antibiotics (Basel). 2020;9(12):919.

11. Wang G, Brunel JM, Preusse M, Mozaheb N, Willger SD, Larrouy-Maumus G, et al. The membrane-active polyaminoisoprenyl compound NV716 re-sensitizes Pseudomonas aeruginosa to antibiotics and reduces bacterial virulence. Commun Biol. 2022;5(1):871.

12. O'Callaghan CH, Morris A, Kirby SM, Shingler AH. Novel method for detection of beta-lactamases by using a chromogenic cephalosporin substrate. Antimicrob Agents Chemother. 1972;1(4):283-8.

13. Chalhoub H, Saenz Y, Nichols WW, Tulkens PM, Van Bambeke F. Loss of activity of ceftazidime-avibactam due to MexAB-OprM efflux and overproduction of AmpC cephalosporinase in Pseudomonas aeruginosa isolated from patients suffering from cystic fibrosis. Int J Antimicrob Agents. 2018;52(5):697-701.

14. Borselli D, Lieutaud A, Thefenne H, Garnotel E, Pages JM, Brunel JM, et al. Polyamino-Isoprenic Derivatives Block Intrinsic Resistance of P. aeruginosa to Doxycycline and Chloramphenicol In Vitro. PLoS One. 2016;11(5):e0154490.

15. Cabrini G, Verkman AS. Potential-sensitive response mechanism of diS-C3-(5) in biological membranes. J Membr Biol. 1986;92(2):171-82.

16. Wang G, Brunel JM, Rodriguez-Villalobos H, Bolla JM, Van Bambeke F. The polyamino-isoprenyl potentiator NV716 revives disused antibiotics against Gram-negative bacteria in broth, infected monocytes, or biofilms, by disturbing the barrier effect of their outer membrane. Eur J Med Chem. 2022;238:114496.

17. Sarrazin M, Martin BP, Avellan R, Gnawali GR, Poncin I, Le Guenno H, et al. Synthesis and Biological Characterization of Fluorescent Cyclipostins and Cyclophostin Analogues: New Insights for the Diagnosis of Mycobacterial-Related Diseases. ACS Infect Dis. 2022;8(12):2564-78.

18. Barelier S, Avellan R, Gnawali GR, Fourquet P, Roig-Zamboni V, Poncin I, et al. Direct capture, inhibition and crystal structure of HsaD (Rv3569c) from M. tuberculosis. FEBS J. 2023;290(6):1563-82.

19. Madani A, Mallick I, Guy A, Crauste C, Durand T, Fourquet P, et al. Dissecting the antibacterial activity of oxadiazolone-core derivatives against Mycobacterium abscessus. PLoS One. 2020;15(9):e0238178.

20. Madani A, Ridenour JN, Martin BP, Paudel RR, Abdul Basir A, Le Moigne V, et al. Cyclipostins and Cyclophostin Analogues as Multitarget Inhibitors That Impair Growth of Mycobacterium abscessus. ACS Infect Dis. 2019;5(9):1597-608.

21. Cox J, Hein MY, Luber CA, Paron I, Nagaraj N, Mann M. Accurate proteome-wide label-free quantification by delayed normalization and maximal peptide ratio extraction, termed MaxLFQ. Mol Cell Proteomics. 2014;13(9):2513-26.

22. Cox J, Mann M. MaxQuant enables high peptide identification rates, individualized p.p.b.-range mass accuracies and proteome-wide protein quantification. Nat Biotechnol. 2008;26(12):1367-72.

23. Cox J, Neuhauser N, Michalski A, Scheltema RA, Olsen JV, Mann M. Andromeda: a peptide search engine integrated into the MaxQuant environment. J Proteome Res. 2011;10(4):1794-805.

24. The UniProt C. UniProt: the universal protein knowledgebase. Nucleic Acids Res. 2017;45(D1):D158-D69.

25. Tusher VG, Tibshirani R, Chu G. Significance analysis of microarrays applied to the ionizing radiation response. Proc Natl Acad Sci U S A. 2001;98(9):5116-21.

26. Deutsch EW, Bandeira N, Sharma V, Perez-Riverol Y, Carver JJ, Kundu DJ, et al. The ProteomeXchange consortium in 2020: enabling 'big data' approaches in proteomics. Nucleic Acids Res. 2020;48(D1):D1145-D52.

27. Perez-Riverol Y, Bai J, Bandla C, Garcia-Seisdedos D, Hewapathirana S, Kamatchinathan S, et al. The PRIDE database resources in 2022: a hub for mass spectrometry-based proteomics evidences. Nucleic Acids Res. 2022;50(D1):D543-D52.
